# Xueshuantong Improves Functions of Lymphatic Ducts and Modulates Inflammatory Responses in Alzheimer’s Disease Mice

**DOI:** 10.3389/fphar.2021.605814

**Published:** 2021-09-28

**Authors:** Rui Zheng, Yang-mei Huang, Qiang Zhou

**Affiliations:** State Key Laboratory of Chemical Oncogenomics, Guangdong Provincial Key Laboratory of Chemical Genomics, Peking University Shenzhen Graduate School, Shenzhen, China

**Keywords:** Alzheimer’s disease, lymphatic vessels, XST, ceftriaxone, APP/PS1 mice

## Abstract

Recent studies have revealed significant contributions of lymphatic vessels (LVs) to vital functions of the brain, especially related to clearance of waste from the brain and immune responses in the brain. These studies collectively indicate that enhancing the functions of LVs may improve brain functions during brain aging and in Alzheimer’s disease (AD) where LV functions are impaired. However, it is currently unknown whether this enhancement can be achieved using small molecules. We have previously shown that a widely used Chinese herbal medicine Xueshuantong (XST) significantly improves functions and reduces pathology in AD transgenic mice associated with elevated cerebral blood flow (CBF). Here, we show that XST partially rescues deficits in lymphatic structures, improves clearance of amyloid-β (Aβ) from the brain, and reduces the inflammatory responses in the serum and brains of transgenic AD mice. In addition, we showed that this improvement in the lymphatic system occurs independently of elevated CBF, suggesting independent modulation and limited interaction between blood circulation and lymphatic systems. Moreover, XST treatment leads to a significant increase in GLT-1 level and a significantly lower level of MMP-9 and restores AQP4 polarity in APP/PS1 mice. These results provide the basis for further exploration of XST to enhance or restore LV functions, which may be beneficial to treat neurodegenerative diseases or promote healthy aging.

## Introduction

New drugs are urgently needed for Alzheimer’s disease (AD), especially those that do not target amyloid-β (Aβ) and tau since a variety of those drugs have shown minimal effectiveness in the clinical trials (Polanco et al., 2018; Zhu et al., 2019). Reduced cerebral blood flow (CBF) is reported in both AD patients and mice and has been linked to various pathologies in AD (Kisler et al., 2017; Kametani and Hasegawa, 2018; Vogelsang et al., 2018). Limited evidence suggests that improving CBF leads to improved functions and reduced pathology in AD transgenic mice (Kisler et al., 2017; Bracko et al., 2019). Recent studies also highlight the important contributions of lymphatic vessels (LVs) to the impaired brain functions in aging and in AD (van der Kleij et al., 2018; Da Mesquita et al., 2018a; Da Mesquita et al., 2018b). Treating aged mice with vascular endothelial growth factor C (VEGF-C) enhances meningeal lymphatic drainage of macromolecules from the cerebrospinal fluid and improves brain perfusion and learning/memory functions (Karaman et al., 2018; Patel et al., 2019). Therefore, the ability to correct or compensate for the deficits in LV structure and functions and restore the brain clearance system is of great interest and importance for promoting healthy aging and treating neurodegenerative diseases (such as AD). No small molecule compound is known to modulate the structure/function of LVs. Given the importance of the lymphatic system, such small molecules may have significant therapeutic potentials.

LVs run within dural leaflets, on the inside surface of the dura, or in the subarachnoid space with the cortical veins (Thomas et al., 2019). Extensive meningeal LV network serves to clear macromolecules and regulate immune cell trafficking in the brain (Tarasoff-Conway et al., 2016; Bálint et al., 2019). This LV system drains interstitial fluids containing wastes from tissues and supports immune surveillance of the brain *via* recirculating immune cells (Venero Galanternik et al., 2016). In the lymphatic network, fluid, cells, and macromolecules are first absorbed by blind-ended lymphatic capillaries (initial LVs) (Klotz et al., 2015; Dave et al., 2018), proceed *via* collector LVs and lymph nodes, and end in the thoracic duct and right lymphatic trunk where lymphatic fluids are delivered into subclavian veins in the neck region (Aspelund et al., 2016; Bálint et al., 2019). Cerebrospinal fluid (CSF) enters the venous system *via* arachnoid granulations (Kortela et al., 2017), while macromolecules and immune cells in the CSF are transported along the dural LVs into the lymph nodes and extracranial systemic circulation and may induce immune responses (Shechter et al., 2013; Schläger et al., 2016). But how exactly blood circulation and lymphatic functions may interact with each other is poorly understood.

Function of LVs is compromised during aging and in neurodegenerative diseases, such as AD (Mesquita et al., 2018; Da Mesquita et al., 2018b; Sun et al., 2018b). Impaired meningeal lymphatic function slows down the perivascular influx of macromolecules into the brain and efflux of macromolecules from the interstitial fluid and induces cognitive impairment in mice (Guimaraes-Souza et al., 2019; Patel et al., 2019). Thus, LVs play an essential role in regulating homeostasis in the brain by draining macromolecules from cerebrospinal fluid (CSF) and interstitial fluid (ISF) into the cervical lymph nodes (CLNs) (Brouillard et al., 2014). Accumulation of Aβ inside LVs of meninges of AD patients or AD transgenic mouse brains may contribute to AD progression (Da Mesquita et al., 2018b; Sun et al., 2018b). Disruption of LVs in transgenic AD mice promotes Aβ deposition in the meninges which resembles human meningeal pathology, and this disruption aggravates parenchymal Aβ accumulation (Kyrtsos and Baras, 2014; Mesquita et al., 2018).

In our previous study, we showed that a widely used Chinese herbal medicine Xueshuantong (XST) significantly elevates CBF, improves memory functions, and reduces AD-relevant pathology in AD transgenic mice (APP/PS1) (Huang et al., 2018). Injected XST (lyophilized) (Supplementary Table S1) is a standardized product extracted from the rhizome comminution of *Panax notoginseng* [Araliaceae*; Panax notoginseng (*Burkill) F.H.Chen] and has been approved in treating stroke by the State Food and Drug Administration in China since 2002 (Wang et al., 2018). XST contains Notoginsenoside R1 (11.1%), Ginsenoside Rg1 (48.1%), Ginsenoside Re (5.5%), Ginsenoside Rb1 (27.8%), and Ginsenoside Rd (1.3%) (Supplementary Figure S1) (Yao et al., 2017; Wang et al., 2018). It is reported that XST significantly reduces inflammatory responses through NRF-2/Keap1 Pathway (Du et al., 2015; Wang et al., 2018), but whether this effect is mediated by elevated CBF is not known. In this study, we examined whether the same XST treatment as we used previously alters LV structure in APP/PS1 mice, and if so, whether such changes are associated with improved brain clearance of Aβ from the brain parenchyma. We also examined whether XST affects inflammatory responses in AD mice. Moreover, we examined whether enhanced CBF affects LVs in order to understand potential interactions between blood circulation and lymphatic system.

## Materials and Methods

*Animals.* APPswe/PSEN1dE9 mice with a C57BL/6 background were obtained from the Jackson Laboratory. APP/PS1 mice and wild-type littermate mice were genotyped by PCR analysis of genomic DNA. All experiments have been approved by the Peking University Shenzhen Graduate School Animal Care and Use Committee (Permit Number: AP0011) and were in accordance with the ARRIVE guidelines on the Care and Use of Experimental Animals. Male and female animals were fed separately and housed in a group of 4–5. The total number of APP/PS1 mice is 72 and wild-type mice is 32. Males and females were used equally in all studies. We also found no impact of sex on the pathology of lymphatic drainage at baseline (Supplementary Figure S4), consistent with previous studies (Da Mesquita et al., 2018b). All mice were maintained under standard laboratory conditions at 22 ± 2°C, with 50 ± 10% relative humidity, and on a 12 h light/dark cycle, with food and water available ad libitum. All mice were 11 months old.

*Drugs.* Xueshuantong (XST) (batch number: Z45021769) was obtained from Wuzhou Pharmaceutical Co., Ltd., China. The manufacturing technology was based on “Pharmacopoeia of China 2005.” The rhizome comminution of *Panax notoginseng* [Araliaceae*; Panax notoginseng (*Burkill) F.H.Chen] was extracted by 60% ethanol under reflux three times (3 h per time). The extracting solution was merged for decompressing concentration till there was no alcohol taste and it was further subjected to a D101 macroporous absorption resin column eluted with water, 80% ethanol. The 80% ethanol extract was dried under vacuum to obtain XST (Wang et al., 2015). Previous studies have fully studied the main compositions and contents of this preparation (Yao et al., 2017; Zhang et al., 2020). The HPLC fingerprint shows that it contains Notoginsenoside R1 (11.1%, PubChem CID: 441934), Ginsenoside Rg1 (48.1%, PubChem CID: 441923), Ginsenoside Re (5.5%, PubChem CID: 441921), Ginsenoside Rb1 (27.8%, PubChem CID: 9898279), and Ginsenoside Rd (1.3%, PubChem CID: 11679800) (Yao et al., 2017). Freeze-dried powder of XST was dissolved in saline and administrated intraperitoneally (100 mg/kg), and saline injection was used as control. Dose and route of Xueshuantong administration used in the present study were based on a commonly used dosage of Xueshuantong in clinical practice and previous preclinical studies (Guo et al., 2018; Huang et al., 2018; Li et al., 2020). For experiments with 30-day injection (i.p.), 15 daily injections with a break of days were used. For experiments with 15-day injection (i.p.), daily injection was used. For examining short-term (5 d) effects, one injection was used.

Ceftriaxone (Cef) (Sigma) was dissolved in saline and administrated intraperitoneally (20 mg/kg), and saline injection was used as control. For experiments with 15-day injection (i.p.), daily injections were used. All tests were performed 24 h after the end of the last treatment.

*Meninge/Tissue Collection and Processing*. Mice were anesthetized with pentobarbital sodium (1%, 30 mg/kg, i.p.) and perfused with 0.1 M PBS. After removing mandibles and skull rostral to the maxillae, the top of the skull was removed with surgical scissors. Whole-mount meninges were fixed while still attached to the skull cap using 4% paraformaldehyde (PFA) for 24 h at 4°C. Fixed meninges (dura mater and arachnoid) were carefully dissected from the skullcaps with #5 forceps (Fine Science Tools) and kept in PBS at 4°C until further use. Fixed brains were then washed with PBS, cryoprotected with 30% sucrose, and frozen in Tissue-Plus OCT compound (Thermo Fisher Scientific). Fixed and frozen brain sections (30 μm thickness) were cut on a cryostat (Leica) and kept in PBS at 4°C. Frozen lymph nodes were sliced to 30 μm thickness and collected onto gelatin-coated Superfrost Plus slides (Thermo Fisher Scientific) and stored at −20°C.

*Immunohistochemistry*. Tissue was rinsed in PBS and washed with PBS and 0.5% Triton X-100 for 10 min, followed by incubation in PBS and 0.5% Triton X-100 containing 0.5% serum (goat or chicken) and 0.5% bovine serum albumin (BSA) for 1 h at room temperature. Then sections were incubated with primary antibodies: anti-CD31 (eBioscience, clone 390, 1:100), anti-Lyve-1 (eBioscience, clone ALY7, 1:200), anti-CD3e (eBioscience, clone 17A2, 1:500), anti-Aβ (Abcam, ab10148, 1:500), anti-GFAP (Abcam, ab4674, 1:500), and anti-Iba1 (Abcam, ab178847, 1:500), overnight at 4°C in PBS containing 1% BSA and 0.5% Triton X-100. Whole mounts and sections were then washed three times (5 min each) at room temperature in PBS, followed by incubation with Alexa Fluor 488/594/647 chicken/goat anti-rabbit/goat IgG antibodies (Invitrogen, 1:1,000) for 1 h at room temperature in PBS with 1% BSA and 0.5% Triton X-100. After 5 min in DAPI (Invitrogen, 1:10,000), whole-mount sections were washed with PBS and mounted with Aqua-Mount (Lerner). Preparations were stored at 4°C for no more than 1 week before images were acquired on a Nikon AR1 confocal system (Nikon Corporation).

*Enzyme-Linked Immunosorbent Assay (ELISA)*. ELISAs were performed to detect IL-10, IL-6, and IL-1β using specific kits (Boshide Bio, China). All blood samples of APP/PS1 or C57 mice, control, or XST-treated group were harvested and cryopreserved at −80°C until analysis. All kits were used according to the manufacturer’s suggested protocol.

*Western Blot.* As our previously described (Gao et al., 2019), tissues were collected, homogenized in RIPA buffer (Beyotime Biotechnology, China) containing 1 mM PMSF (Sigma), 25 μM leupeptin (Sigma), and 1 μg/ml aprotinin (Sigma), and centrifuged at 4°C for 0.5 h at 10,000 g, with the supernatant collected. Total protein lysates made from hippocampus or entire brain were mixed with SDS gel-loading buffer and heated for 5 min at 100°C. Samples (15 μg protein in each group) were separated on 10% SDS-PAGE gels (Invitrogen, Carlsbad, CA, United States) and transferred to polyvinylidene difluoride membranes (Millipore, Bedford, MA, United States). Membranes were blocked for 1 h at room temperature with 5% nonfat milk in TBST (TBS containing 0.05% Tween 20) and then probed with a specific primary antibody (anti-AQP4, 1:1000, Cat. No. ab46182, Abcam, United Kingdom; anti-GLT1, 1:1000, Cat. No. ab41621, Abcam, United Kingdom; anti-MMP-9, 1:500, Cat. No. ab38898, Abcam, United Kingdom; anti-GAPDH, 1:10000, Cat. No. ab8245, Abcam, United Kingdom, anti-α-Tubulin, 1:10000, Cat. No. 5335T, CST, United Kingdom) overnight at 4°C. A horseradish peroxidase- (HRP-) conjugated secondary antibody (anti-mouse or anti-rabbit, NeoBioscience) was added for 2 h at room temperature. Immunopositive AQP4, GLT1, MMP-9, and GAPDH bands were scanned and densitometrically analyzed by automated ImageJ software (NIH Image, Version 1.61), and their total protein densities were expressed relative to GAPDH or α-Tubulin signals.

*Image Analysis*. Images were acquired with a Nikon AR1 confocal system (Nikon Corporation) using the LAS AF software. All images were acquired with a 1,024 × 1,024 pixel resolution. Quantitative analysis was performed using FIJI software (NIH). Diameters of LVs and blood vessels (BVs) of the meninges were quantified using ImageJ. A standard length (100 μm) was first marked on the images to be analyzed, then the structures to be analyzed were selected, and sizes of these identified structures were automatically measured by ImageJ. Percentage of T-cells was determined by counting the number of T-cells within the superior sagittal sinus (SSS) or transverse sinus (TS). T-cell density was calculated by dividing the number of T lymphocytes by the area of the field. The density of Aβ was calculated by dividing the number of Aβ plaques by the area of field.

*AQP4 Expression Polarity Measurement*. To calculate the AQP4 polarity for each image, each color channel was processed separately with each image thresholded uniformly at two different levels, a high- and a low-stringency threshold. The low-stringency threshold defined the overall area of AQP4-immunoreactivity while the high-stringency threshold defined the area of intense AQP4-immunoreactivity that in control mice is localized to perivascular endfeet. The ratio of low- and high-stringency area was used to generate an arbitrary value defined as “AQP4 polarity” (Supplementary Figure S3) (Wang et al., 2012). The higher the AQP4 polarity was, the greater the proportion of immunoreactivity was restricted to the perivascular regions, while the lower the proportion, the more the evenly distributed immunoreactivity was between the perivascular endfeet and the soma.

*Two-Photon Time-Lapse Imaging of Blood Flow.* As previously described (Huang et al., 2018), mice were injected with Cef or saline daily for 15 days, and microvessels were imaged on day 15 using two-photon imaging using Olympus BX 51 microscope. To measure blood flow in the microvessels (diameter 7–15 μm or 40–60 μm), 25 μl of 10% fluorescein sodium salt (Sigma-Aldrich) was injected through the mouse tail vein 1 h prior to imaging. Several frame images with different zooms were collected to facilitate the identification of the same set of vessels by using landmarks in these images. The speed of the movement of the RBCs was calculated from the strips in the line scan images. The instantaneous velocity of RBCs is calculated as V = Δy/Δt; flux = 1/Δt; linear density = 1/Δy.

*Statistical Analysis*. Data were analyzed using GraphPad Prism 8 software. Unpaired *t*-test, paired *t*-test, and two-way repeated measures ANOVA followed by Bonferroni posttest were used. All results were shown as mean ± SEM. *p* < 0.05 was considered statistically significant.

## Results

### Altered Structures of LVs in APP/PS1 Mice and Improvement by XST Treatment

**FIGURE 1:**
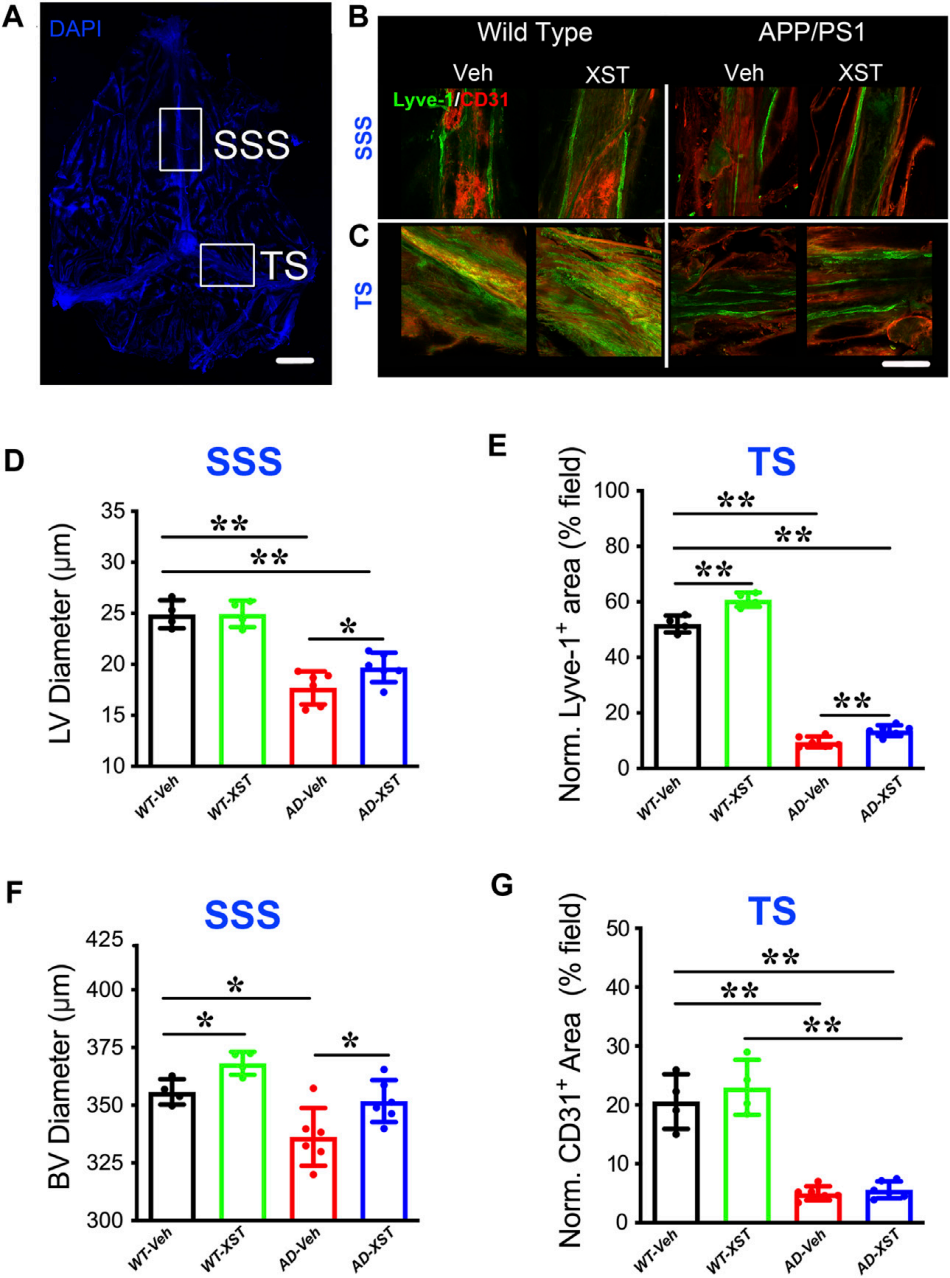
Effects of XST on lymphatic structures. **(A)** Schematic representation of the whole-mount dissection of the dura mater. SSS, superior sagittal sinus; TS, transverse sinus. Scale bar, 2,000 μm. **(B)** Sample images of lymphatic vessels (LVs) (Lyve-1) and blood vessels (BVs) (CD31) in the SSS region, in wild-type and APP/PS1 mice treated with either XST or Vehicle (Veh). C57-Veh (*n* = 4 mice), C57-XST (*n* = 4 mice), AD-Veh (*n* = 6 mice), AD-XST (*n* = 6 mice). Scale bar, 200 μm. **(C)** Sample images of lymphatic ducts (Lyve-1) and BVs (CD31) in the TS region, in wild-type and APP/PS1 mice treated with either XST or Veh. Scale bar, 200 μm. **(D)** Quantification of the diameter of lymphatic ducts in mice treated with XST or Veh in the SSS region (F _(1, 16)_ = 87.18, C57 vs. AD, *p* < 0.0001; AD-Veh vs. AD-XST, *p* = 0.034, two-way ANOVA with Bonferroni’s multiple comparisons). **(E)** Quantification of the area occupied by lymphatic ducts in mice treated with XST or Veh in the TS region (F _(1, 16)_ = 36.92, C57 vs. AD, *p* < 0.0001; C57-Veh vs. C57-XST, *p* < 0.001; AD-Veh vs. AD-XST, *p* = 0.0454, two-way ANOVA with Bonferroni’s multiple comparisons). **(F)** Quantification of the diameter of BVs in mice treated with XST or Veh in the SSS region (F _(1, 16)_ = 22.18, C57 vs. AD, *p* = 0.0002; C57-Veh vs. C57-XST, *p* = 0.049; AD-Veh vs. AD-XST, *p* = 0.0303, two-way ANOVA with Bonferroni’s multiple comparisons). **(G)** Quantification of the area occupied by BVs in mice treated with XST or Veh in the TS region (F _(1, 16)_ = 141.4, C57 vs. AD, *p* < 0.0001; two-way ANOVA with Bonferroni’s multiple comparisons). Data are mean ± SEM. *p < 0.05; **p < 0.01.

By using immunostaining, we imaged the structure of LVs in both APP/PS1 and WT mice, treated with XST or Vehicle (Veh). Two regions were examined, SSS and TS (Figure 1A), where LVs have been shown to be altered in both AD and during aging (Mesquita et al., 2018). In the SSS region, diameters of LVs in APP/PS1 mice were significantly smaller than those in WT mice (Figure 1B; Lyve-1^+^; Figure 1D; *p* < 0.01), consistent with previous results on defected LVs in AD brains (Da Mesquita et al., 2018b). The XST-treated APP/PS1 group, compared to the Veh-treated group, had significantly larger LV diameters, a change not observed in WT mice (Figure 1D; *p* < 0.05). Since LV diameters cannot be reliably measured in the TS region, we measured areas positive for Lyve-1 in TS (% of entire field) (Patel et al., 2019). We found a significant reduction in the Lyve-1^+^ area in the APP/PS1 mice compared to WT mice (Figures 1C,E; *p* < 0.01), while these areas were significantly larger in XST-treated mice (both APP/PS1 and WT) (Figures 1C,E; *p* < 0.01).

In addition to LVs, we also measured BVs attached to the meninges. In the SSS region of both APP/PS1 and WT mice (Figure 1B; CD31^+^), we found a significant reduction in the diameters of CD31^+^ BVs in APP/PS1 mice compared to WT mice (Figures 1B,F; *p* < 0.05), while XST-treated group exhibited significantly larger diameters in both APP/PS1 and WT mice (Figures 1B,F; *p* < 0.05). In the TS region, there was a significant reduction in the CD31^+^ area in APP/PS1 mice compared to WT (Figures 1C,G; *p* < 0.01), but no difference was seen between XST- and Veh-treated groups in either WT or APP/PS1 mice. Thus, the impacts of XST on BVs are brain region-dependent.

### Reduced Aβ Presence in LVs in XST-Treated APP/PS1 Mice

**FIGURE 2:**
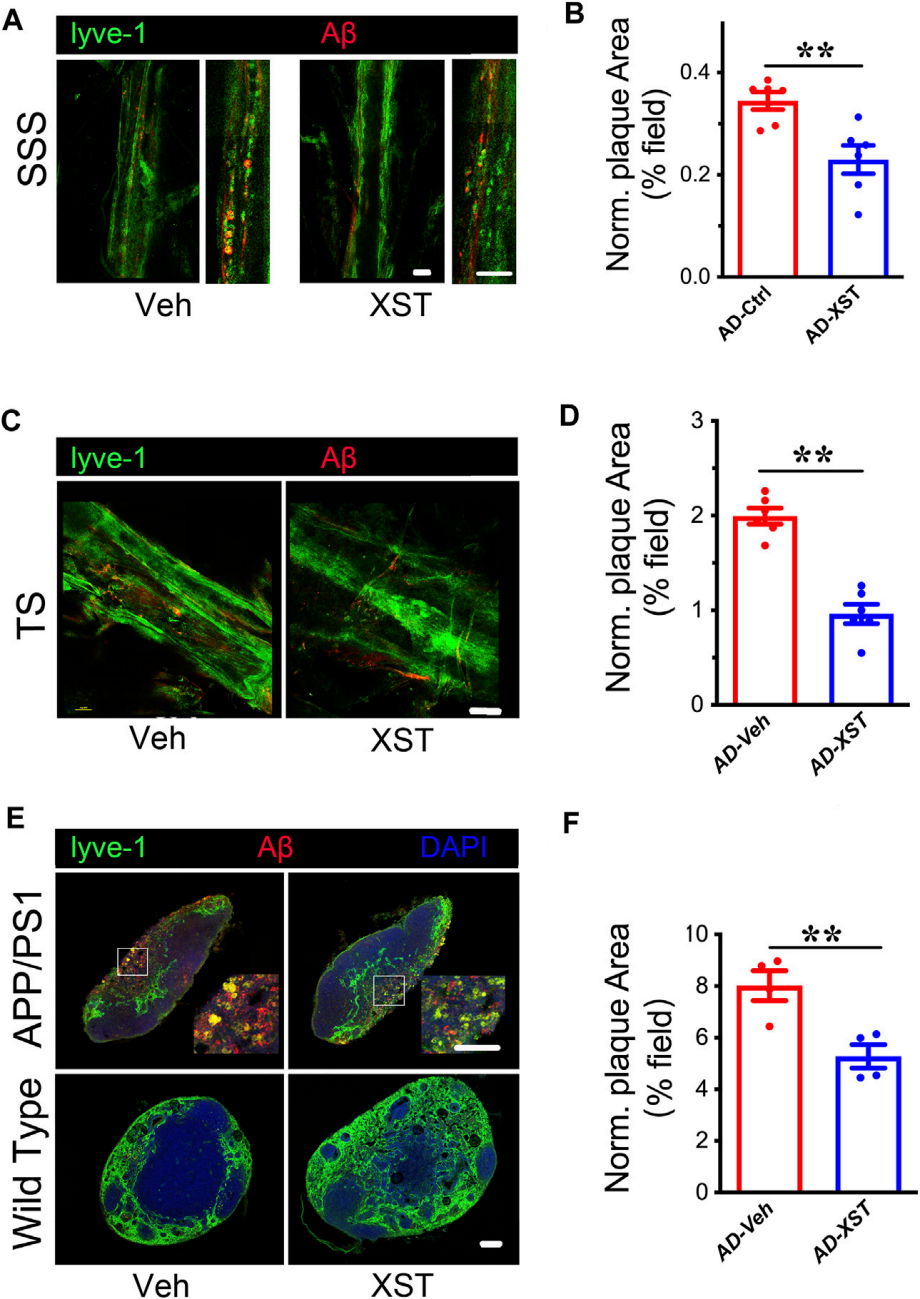
XST improves Aβ removal from brains of APP/PS1 mice. **(A)** Impact of XST on the removal of Aβ *via* the brain lymphatic ducts in APP/PS1 mice, in the SSS and TS regions. Red, Aβ; green, Lyve-1. Scale bars, 100 μm. **(B)** Quantification of plaque area in the SSS region in XST- or Veh-treated group (*p* = 0.0056, *t*-test, *n* = 6 mice per group). **(C)** Impact of XST on Aβ removal *via* the brain lymphatic ducts in APP/PS1 mice, in the TS regions. Red, Aβ; green, Lyve-1. Scale bar, 100 μm. **(D)** Quantification of plaque area in the TS region XST- or Veh-treated group (*p* = 0.0067, *t*-test, *n* = 4 mice per group). **(E)** Sample images if Aβ staining in the lymph nodes of the neck. Red, Aβ; green, Lyve-1; blue, DAPI. Scale bar, 200 μm (low). Scale bar, 100 μm (high). **(F)** Quantification of plaque area in the lymph nodes of the neck region in XST- or Veh-treated APP/PS1 mice (*p* = 0.0098, *t*-test, *n* = 4 mice per group). Data are mean ± SEM. *p < 0.05; **p < 0.01.

Previous studies indicated enhanced clearance ability with enlarged LVs (Da Mesquita et al., 2018a; Sun et al., 2018b). To test whether this is the case with XST, we examined Aβ clearance in the brain after XST or Veh treatment by staining Aβ in brain sections containing either SSS or TS region. We found that, in the XST-treated group, Aβ-positive areas are significantly smaller (Figures 2A,B; *p* < 0.01), suggesting a more effective removal of Aβ from LVs with XST treatment. We also found a significantly smaller Aβ plaque area in the TS region of XST-treated APP/PS1 mice (Figures 2C,D; *p* < 0.01). Due to the absence of Aβ in WT mouse brains, this analysis was not performed.

Macromolecules in CSF are transported mainly along the dural LVs into lymph nodes of the neck and extracranial systemic circulation (Kisler et al., 2017; Da Mesquita et al., 2018b). It was reported that Aβ is drained from TS into the lymph nodes along the side of the neck (Louveau et al., 2017). We then examined Aβ plaques in regions where LVs merge with BVs in the lymph node (Figure 2E). A significantly smaller Aβ area was seen in the XST-treated compared to Veh-treated APP/PS1 mice (Figures 2E,F; *p* < 0.01). All together, these results indicate a reduced Aβ plaque size consistent with our previous findings (Huang et al., 2018) and suggest that XST enhances Aβ clearance capacity in APP/PS1 mice.

### Reduced Inflammatory Responses With XST Treatment

**FIGURE 3:**
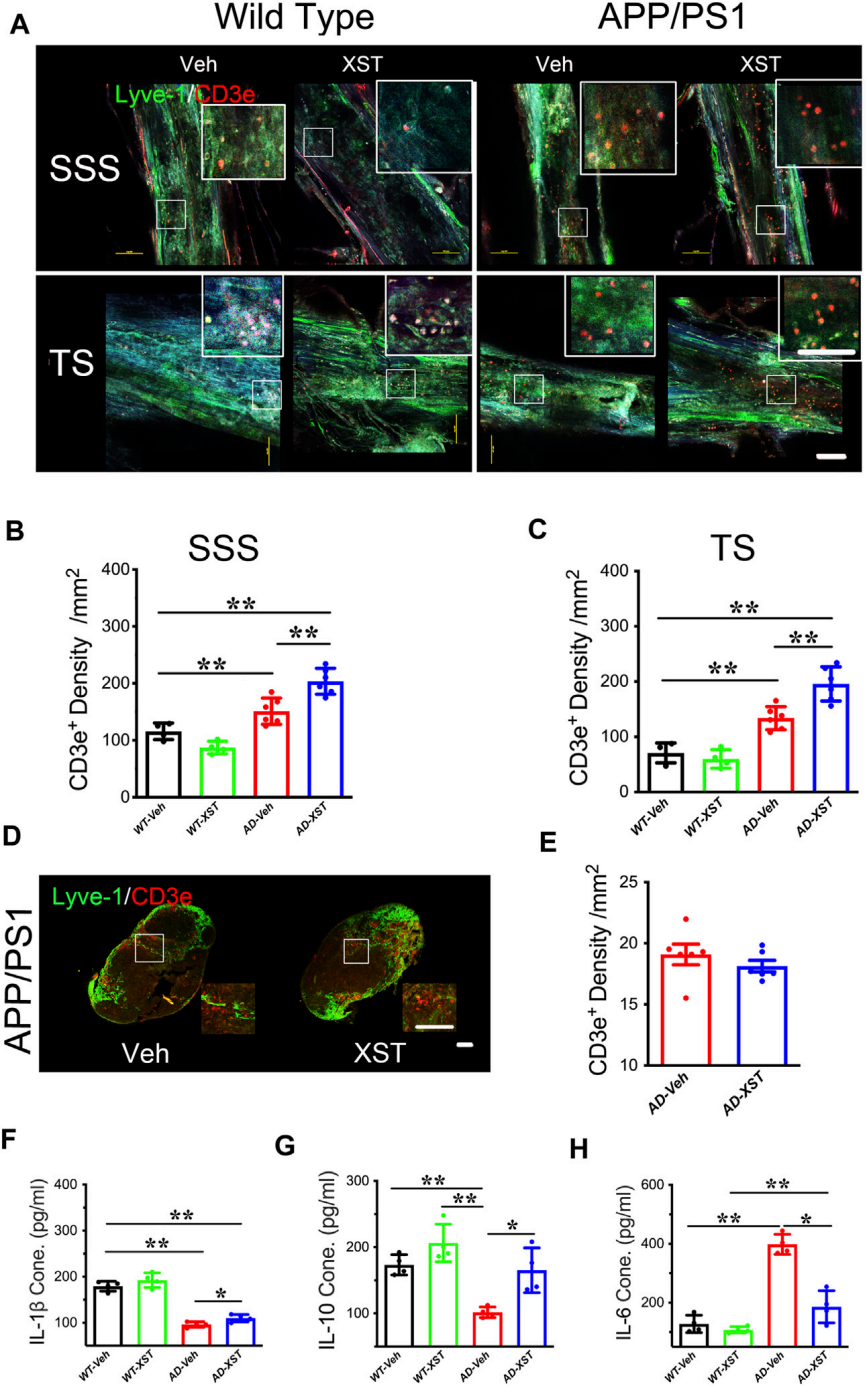
Effect of XST on inflammatory responses in the brains of APP/PS1 mice. **(A)** Sample images of lymphatic ducts (Lyve-1, green) and T-cells (CD3e, red) in SSS region and TS region, in WT and APP/PS1 mice treated with either XST or Veh. Scale bar, 100 μm. **(B)** Quantification of T-cell density in lymphatic ducts of the SSS region in WT and APP/PS1 mice treated with XST or Veh (F _(1, 16)_ = 70.96, C57 vs. AD, *p* < 0.0001; AD-Veh vs. AD-XST, *p* = 0.0017, two-way ANOVA with Bonferroni’s multiple comparisons). **(C)** Quantification of the T-cell density in lymphatic ducts of the TS region in WT and APP/PS1 mice treated with XST or Veh (F _(1, 16)_ = 86.28, C57 vs. AD, *p* < 0.0001; AD-Veh vs. AD-XST, *p* = 0.0019, two-way ANOVA with Bonferroni’s multiple comparisons). **(D)** Sample images of T-cells in the lymph nodes of the neck. T-Cells (CD3e, red); lymph nodes (Lyve-1, green). Scale bar, 200 μm (low). Scale bar, 100 μm (high). **(E)** Quantification of T-cells density in the lymph nodes of the neck region in XST- or Veh-treated APP/PS1 mice (*p* = 0.3483, *t*-test, *n* = 6 mice per group). Quantitative measurement of IL-1β **(F)** (F _(1, 12)_ = 232.5, C57 vs. AD, *p* < 0.0001; AD-Veh vs. AD-XST, *p* = 0.0279, two-way ANOVA with Bonferroni’s multiple comparisons); IL-6 **(G)** (F _(1, 12)_ = 94.89, C57 vs. AD, *p* < 0.0001; AD-Veh vs. AD-XST, *p* = 0.014, two-way ANOVA with Bonferroni’s multiple comparisons); and IL-10 **(H)** (F _(1, 12)_ = 22.63, C57 vs. AD, *p* = 0.0005; AD-Veh vs. AD-XST, *p* = 0.0155, two-way ANOVA with Bonferroni’s multiple comparisons). Levels in serum in WT and APP/PS1 mice (*n* = 4 mice per group). Data are mean ± SEM. IL: interleukin. *p < 0.05; **p < 0.01.

Another reported consequence of enhanced LV structure/function is an improved inflammatory response shown as higher T-cell density in the SSS region (Kim and Song, 2017). To examine this, we first counted the number of CD3e^+^ T-cells in the SSS and TS regions (Figure 3A). Density of CD3e^+^ cells inside LVs was significantly higher in the APP/PS1 mice compared to WT mice (Figures 3A–C; *p* < 0.01 for both regions), suggesting elevated immune responses in APP/PS1 mice (Toly-Ndour et al., 2011; Zhu et al., 2019). A significantly higher density of CD3e^+^ cells was seen in APP/PS1 mice treated with XST, compared to the Veh group (Figure 3B; *p* < 0.01), suggesting a further increase in T-cell density and likely enhanced responses after XST treatment. When the same measurement was repeated in the TS region, we also found a significantly higher density of CD3e^+^ cells in APP/PS1 mice in the XST-treated group compared to the Veh group (Figures 3A,C; *p* < 0.01). These results suggest that XST may promote immune surveillance. However, the CD3e^+^ cell density in neck lump node (CLNs) was not different between the XST- and Veh-treated groups in the APP/PS1 mice (Figures 3D,E), suggesting that T-cell storage in CLNs is not affected by XST.

The above results suggest an increased T-cell density which is expected to reduce inflammation in the brain. We next examined the serum level of IL-1β (a marker of anti-inflammatory response) (Williams et al., 1999). A significantly lower level of IL-1β was seen in APP/PS1 mice compared to WT mice (Figure 3F; *p* < 0.01) and a significantly higher level in the XST-treated APP/PS1 mice (compared to Veh-treated) (Figure 3F; *p* < 0.05). Consistently, serum IL-10 level was significantly higher in the XST-treated APP/PS1 mice than in Veh-treated APP/PS1 mice (Figure 3G; *p* < 0.05). On the other hand, serum IL-6 level (a proinflammation cytokine) was significantly higher in the APP/PS1 mice compared to WT mice (Figure 3H; *p* < 0.01) and significantly lower in the XST-treated APP/PS1 mice compared to Veh group (Figure 3H; *p* < 0.05). In addition, we also found a significantly lower IL-6 level in the brain in the XST-treated APP/PS1 mice (Supplementary Figure S2C; *p* < 0.05) and a significantly higher IL-10 level in the XST-treated APP/PS1 mice than in Veh-treated APP/PS1 mice (Supplementary Figure S2D; *p* < 0.01). Thus, these results suggest that the inflammation level is significantly reduced by XST in APP/PS1 mice.

### Cellular Markers of Inflammation in the Brain With XST Treatment

**FIGURE 4:**
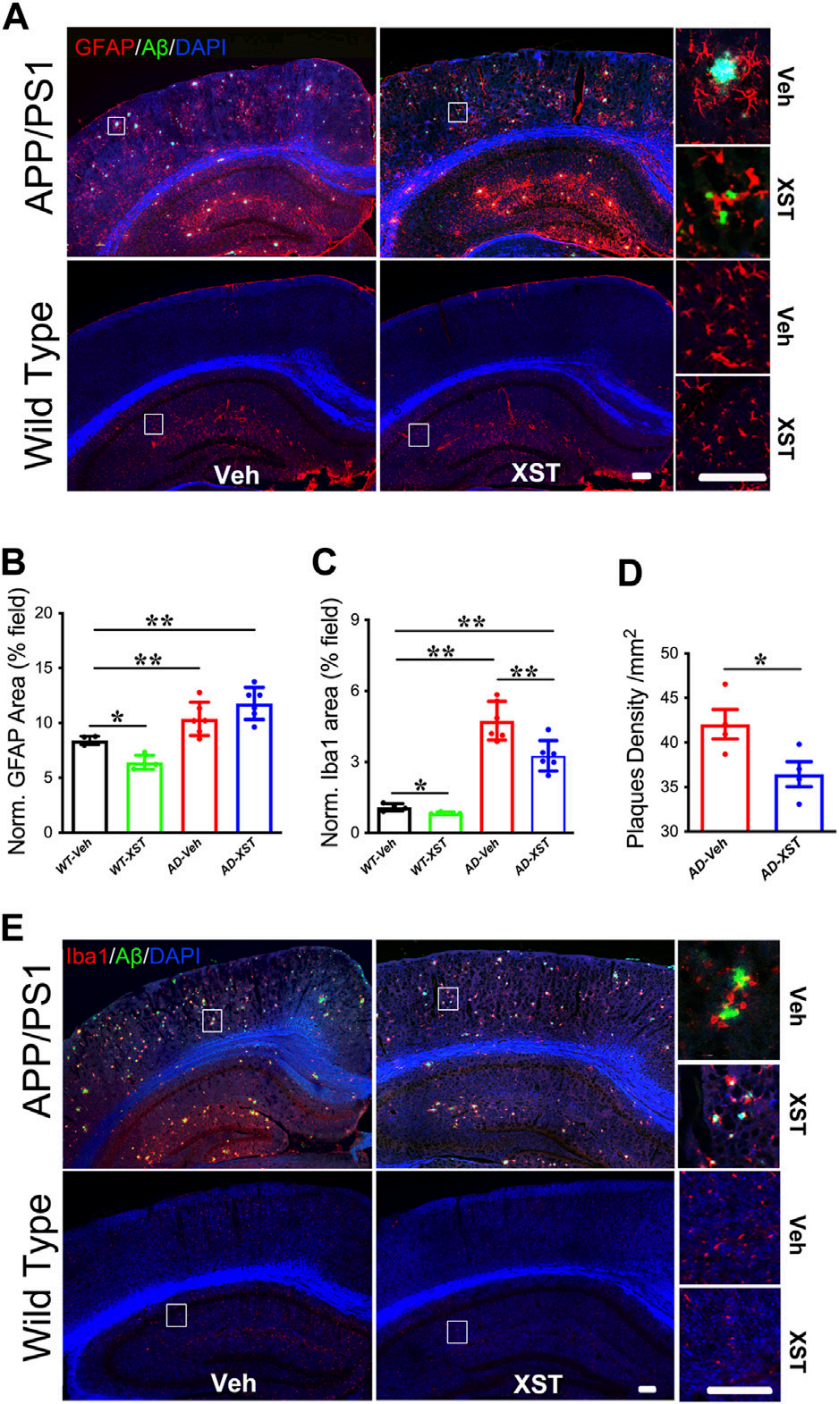
Effect of XST on Aβ removal from the brains of APP/PS1 mice. **(A)** Sample images of amyloid plaques (Aβ, green) and astrocytes (GFAP, red) in the cortex and hippocampus of WT or APP/PS1 mice. Scale bar, 100 μm. **(B)** Quantification of GFAP^+^ area of in the brains of WT and APP/PS1 mice treated with XST or Veh (F _(1, 16)_ = 43.53, C57 vs. AD, *p* < 0.0001; C57-Veh vs. C57-XST, *p* = 0.038, two-way ANOVA with Bonferroni’s multiple comparisons). **(C)** Quantification of Aβ density in the brains of APP/PS1 mice treated with XST or Veh (F _(1, 16)_ = 129.7, C57 vs. AD, *p* < 0.0001; AD-Veh vs. AD-XST, *p* = 0.0028, two-way ANOVA with Bonferroni’s multiple comparisons). **(D)** Quantification of the area of Iba1^+^ in the brain of WT and APP/PS1 mice treated with XST or Veh (*p* = 0.0409, *t*-test, *n* = 4 mice per group). **(E)** Sample images of amyloid plaque (Aβ, green) and microglia (Iba1, red) in the cortex and hippocampus of WT or APP/PS1 mice. Scale bars, 100 μm. Data are mean ± SEM. *p < 0.05; **p < 0.01.

Enhanced responsiveness of both microglia and astrocytes has been widely reported in both AD patients and AD mice (Fakhoury, 2018; Kaur et al., 2019). In the APP/PS1 mice, a larger area occupied by astrocytes (GFAP^+^) was found in APP/PS1 mice (compared to WT mice) (Figures 4A,B; *p* < 0.01), and GFAP^+^ area was not different between XST- and Veh-treated groups (Figures 4A,B). Interestingly, XST-treated WT mice showed a small but significantly smaller GFAP^+^ area (Figures 4A,B; *p* < 0.05). In the same mice, the areas covered by Iba1^+^ cells were significantly larger in APP/PS1 mice (Figures 4C,E; *p* < 0.01) and significantly smaller in APP/PS1 and WT mice treated with XST [Figures 4C,E; *p* < 0.01 (APP/PS1), *p* < 0.05 (wt)]. In addition, we found a significantly smaller Aβ plaque density in XST-treated APP/PS1 mice (Figures 4A,D; *p* < 0.05), consistent with a reduced Aβ level in the previous experiments (Figure 2). Together with the results in Figure 3, XST reduces the activation of astrocytes and microglia which may underlie the altered serum cytokine levels and enhanced clearance of Aβ plaque.

### Time Course of Changes in Astrocytes and Microglia Density After XST Treatment

**FIGURE 5:**
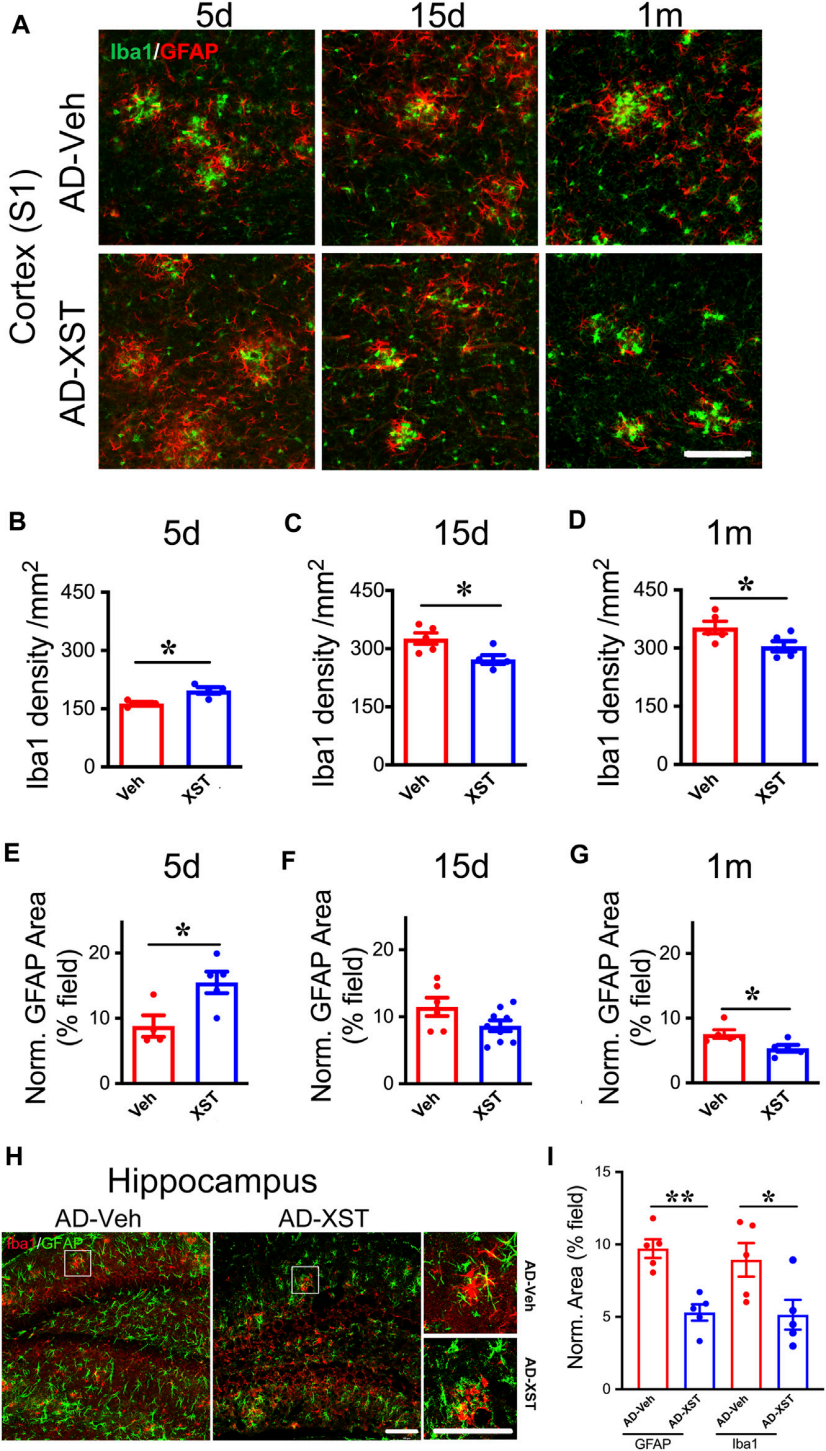
Time course of XST’s impact on microglia and astrocytes in APP/PS1 mice. **(A)** Sample images of microglia (Iba1, green) and astrocytes (GFAP, red) in the cortex (S1) of APP/PS1 mice after treatment with XST for 5 days, 15 days, or 1 month. Scale bar, 100 μm. **(B**–**D)** Quantification of Iba1^+^ density after 5 days **(B)** (*p* = 0.0112, *t*-test, *n* = 4 mice per group), 15 days **(C)** (*p* = 0.0233, *t*-test, AD-Veh, *n* = 5 mice, AD-XST, *n* = 4 mice), and 1 month **(D)** (*p* = 0.0487, *n* = 5 mice per group) of XST treatment in APP/PS1 mice. **(E**–**G)** Quantification of GFAP^+^ area after 5 days **(E)** (*p* = 0.0253, *t*-test, AD-Veh, *n* = 4 mice, AD-XST, *n* = 5 mice), 15 days **(F)** (*p* = 0.0798, *t*-test, AD-Veh, *n* = 6 mice, AD-XST, *n* = 9 mice), and 1 month **(G)** (*p* = 0.0291, *t*-test, *n* = 5 per group) of XST treatment in APP/PS1 mice. **(H)** Sample images of microglia (Iba1, red) and astrocytes (GFAP, green) in the hippocampus of APP/PS1 mice after treatment with XST for 15 days. Scale bar, 100 μm. **(I)** Quantification of GFAP^+^ area (*p* = 0.009, *t*-test, *n* = 5 mice per group) and Iba1^+^ area (*p* = 0.04, *t*-test, *n* = 5 mice per group) in APP/PS1 mice after treatment with XST for 15 days. Data are mean ± SEM. *p < 0.05; **p < 0.01.

In the above analysis, we conducted experiments at one-time point (15 days after XST treatment). To understand the time course of changes in astrocytes and microglia, we analyzed staining of GFAP and Iba1 at 5 days, 15 days, and 1 month in APP/PS1 mouse brains treated with either XST or Veh (Figure 5A). Interestingly, we found a significantly higher density of Iba1 and bigger GFAP-positive area in mice treated with XST for 5 days (Figures 5A,B for Iba1; *p* < 0.05; Figures 5A,E for GFAP; *p* < 0.05). In mice treated with XST for 15 days and 1 month, we found a significantly lower density of Iba1 (Figures 5A,C,D, *p* < 0.05) and the trend towards reduced GFAP-positive area (Figures 5A,F,G) in the cortex (S1). In addition, we also found significantly smaller GFAP-positive and Iba1-positive areas in the hippocampus of mice treated with XST for 15 days (Figures 5H,I, *p* < 0.01, *p* < 0.05). Thus, XST treatment leads to an initial increase in the density of both astrocytes and microglia, which is followed by a decrease in density.

### Elevating CBF Does Not Modulate LVs in APP/PS1 Mice

**FIGURE 6:**
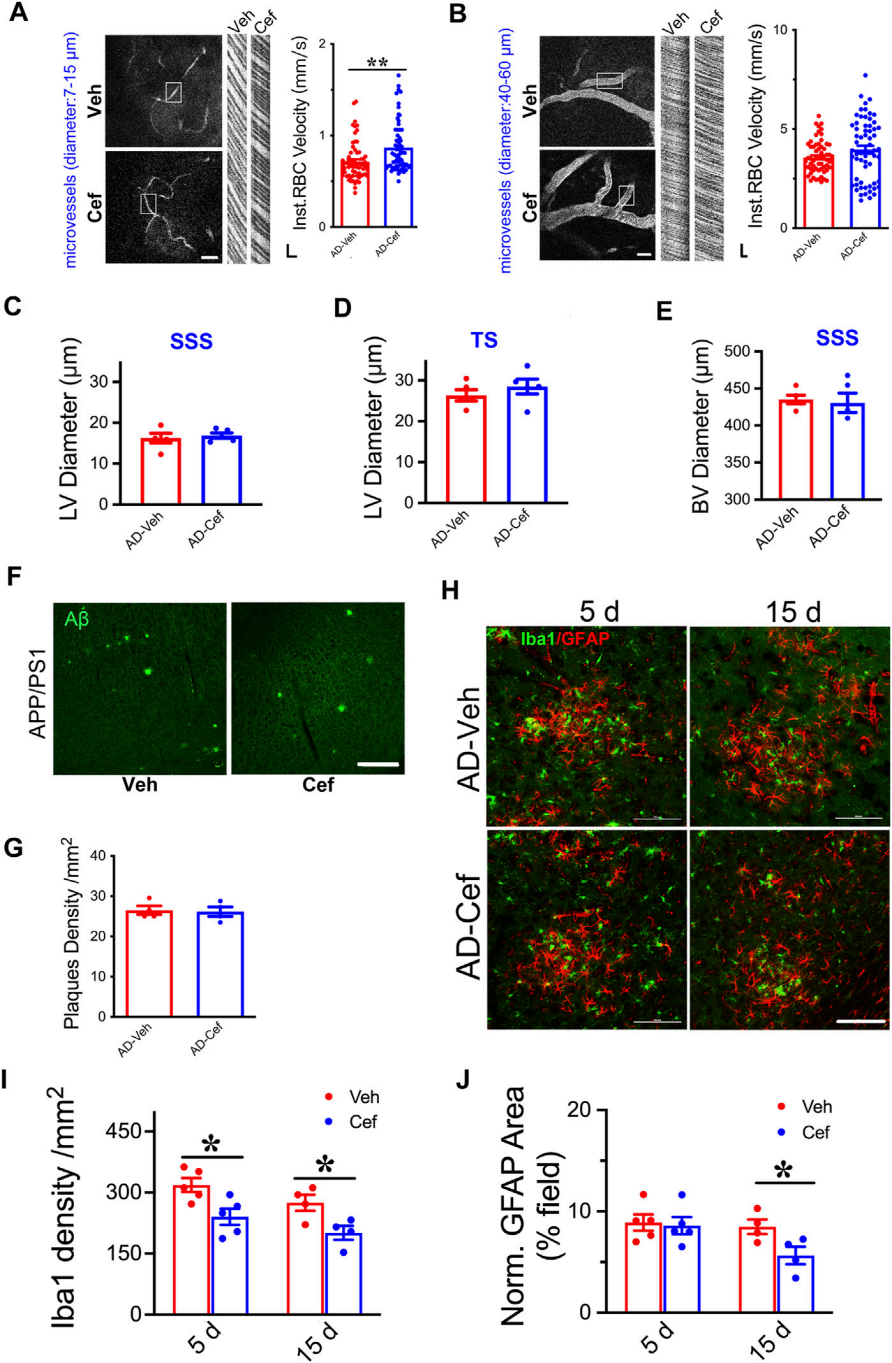
Impacts of Cef on CBF and lymphatic vessel structure in APP/PS1 mice. **(A)** Sample image of small BVs (diameter, 7–15 μm, **left**) and line scan image of blood flow **(right)**. Scale bar, 100 μm **(left)**, 10 ms, and 10 μm **(right)** (*p* = 0.0003, *t*-test, *n* = 64 BVs/4 mice per group). **(B)** Sample image of large BVs (diameter, 40–60 μm, **left**) and line scan image of blood flow **(right)**. Scale bar, 100 μm **(left)**, 10 ms, and 10 μm **(right)** (*p* = 0.0747, *t*-test, *n* = 64 BVs/4 mice per group). **(C)** Quantification of the diameter of lymphatic ducts in mice treated with Cef or Veh in the SSS region (*p* = 0.1316, *t*-test, *n* = 5 mice per group). **(D)** Quantification of the diameter of lymphatic ducts in mice treated with Cef or Veh in the TS region (*p* = 0.1241, *t*-test, *n* = 5 mice per group). **(E)** Quantification of the diameter of BVs in mice treated with Cef or Veh in the SSS region (*p* = 0.3436, *t*-test, *n* = 5 mice per group). **(F)** Sample images of Aβ staining in the cortex of APP/PS1 mice treated with either Cef or Veh. Scale bar, 100 μm. **(G)** Quantification of amyloid plaque density in APP/PS1 mice treated with Cef or Veh (*p* = 0.8199, *t*-test, *n* = 4 mice per group). **(H)** Sample images of Iba1 or GFAP staining in cortex (S1) of APP/PS1 mice treated with either Cef or Veh, for 5 days **(left)** or 15 days **(right)**. Scale bar, 100 μm. **(I)** Quantification of Iba1 density in APP/PS1 mice after treatment with Cef for either 5 days (*p* = 0.0173, *t*-test, *n* = 5 mice per group) or 15 days (*p* = 0.0301, *t*-test, *n* = 4 mice per group). **(J)** Quantification of GFAP^+^ area in APP/PS1 mice after treatment with Cef for either 5 days (*p* = 0.7982, *t*-test, *n* = 5 mice per group) or 15 days (*p* = 0.0447, *t*-test, *n* = 4 mice per group). Data are mean ± SEM. *p < 0.05; **p < 0.01.

The meningeal lymphatic system is necessary for effective clearance of macromolecules from the brain ISF and may serve as a common pathway for effective removal of wastes from brain parenchyma (Plog and Nedergaard, 2018; Chen et al., 2020). We have shown that XST elevated CBF in both APP/PS1 and WT mice (Huang et al., 2018), and thus elevated CBF could drive the improved LV structure/function. Since ceftriaxone (Cef) injection reliably increases CBF (Kortela et al., 2017; Holschneider et al., 2020), we first confirmed this in the APP/PS1 mice using two-photon linescan imaging (Huang et al., 2018). We have focused on small BVs (diameters 7–15 μm) due to their reported alteration in AD (Ahn et al., 2018; Bracko et al., 2019) and used medium-sized vessels as control (diameters 40–60 μm). The velocity of blood flow in small BVs was significantly higher in the Cef-injected APP/PS1 mice compared to Veh-injected (Figure 6A, *p* < 0.01), while no difference was seen in mid-size BVs between these two groups (Figure 6B). We then imaged the structures of LVs and BVs in APP/PS1 mice treated with Cef or Veh. Diameters of LVs in the SSS or TS region were not different between Cef- and Veh-injected groups (Figures 6C,D), while the diameters of BVs in the SSS region were also not different between Cef- and Veh-injected group (Figure 6E). Thus, elevating CBF by Cef injection is not sufficient to alter LV structure in APP/PS1 mice.

To understand whether Aβ plaques could be affected by Cef treatment, we measured their density in the primary somatosensory cortex and found no difference between Cef- and Veh-injected APP/PS1 mice (Figures 6F,G), suggesting that elevating CBF by Cef is not sufficient to modulate plaques, in contract to the significant reduction after XST treatment. Since significant alterations in astrocytes and microglia were seen in APP/PS1 mice after XST treatment (Figure 5), we measured the density of these cells in Cef-treated APP/PS1 mice. Microglia density (Iba1) was significantly lower in APP/PS1 mice treated with Cef for either 5 days or 15 days (Figures 6H,I, *p* < 0.05), but the area of astrocyte (GFAP) was lower in the 15-day but not 5-day treatment group (Figures 6H,J). Thus, compared to XST, Cef treatment does not increase astrocytes and microglia density and thus may have a distinct impact on inflammatory responses. Taken together, although elevated CBF is seen after either XST or Cef in APP/PS1 mice, changes in LVs and Aβ plaques were only seen after XST treatment, suggesting that elevated CBF is unlikely to underlie these differential changes in APP/PS1 mice with XST.

### Differential Changes in Protein Levels Induced by XST and Cef Treatment

**FIGURE 7:**
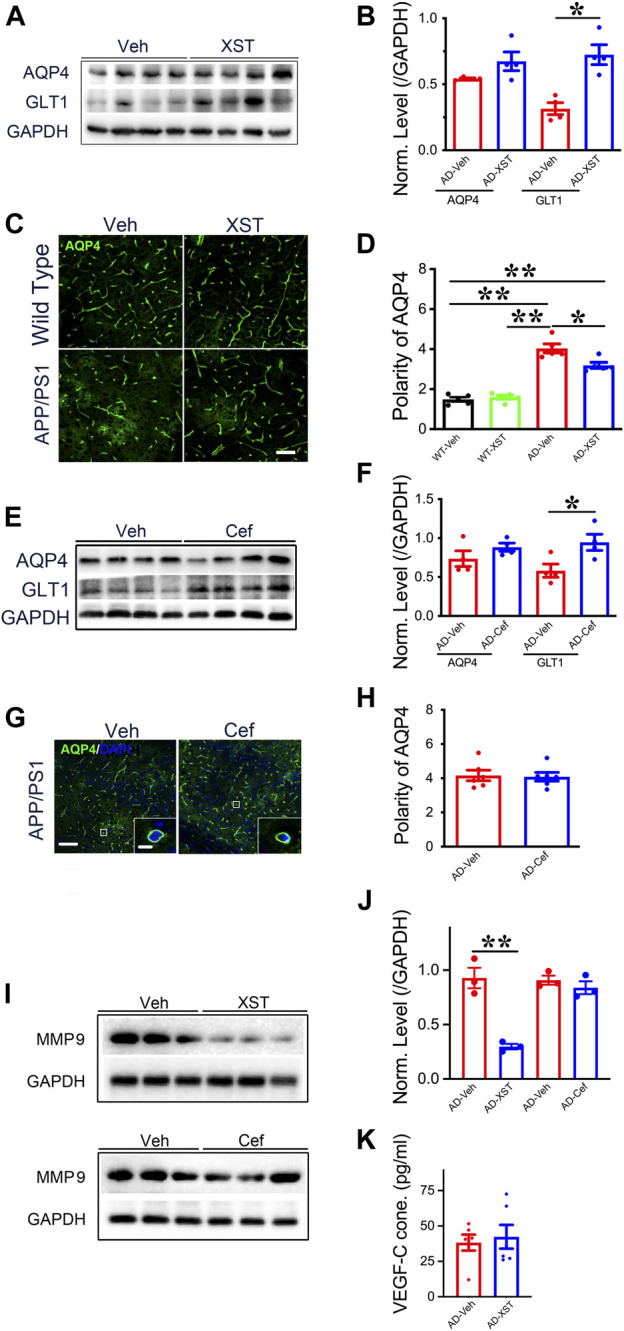
Impacts of XST and Cef on the levels of GLT1, AQP4, and MMP-9 in APP/PS1 mice. **(A)** Sample images of WB analysis of brain AQP4 and GLT1 levels after treatment with XST in APP/PS1 mice. **(B)** Densitometric analysis of protein levels of AQP4 (*p* = 0.1113, *t*-test, *n* = 4 mice per group) and GLT1 (*p* = 0.0035, *t*-test, *n* = 4 mice per group) after treatment with XST, normalized against GAPDH. **(C)** Sample images of AQP4 staining in cortex (S1) of APP/PS1 mice after treatment with XST for 15 days. Scale bar, 50 μm. **(D)** Quantification of AQP4 polarity after XST treatment (F _(1, 16)_ = 183.4, C57 vs. AD, *p* < 0.0001; AD-Veh vs. AD-XST, *p* = 0.0073, two-way ANOVA with Bonferroni’s multiple comparisons, *n* = 5 mice per group). **(E)** Sample images of WB analysis of brain AQP4 and GLT1 levels after treatment with Cef in APP/PS1 mice. **(F)** Densitometric analysis of AQP4 (*p* = 0.1113, *t*-test, *n* = 4 mice per group) and GLT1 (*p* = 0.035, *t*-test, *n* = 4 mice per group) levels after treatment with Cef, normalized to GAPDH. **(G)** Sample images of AQP4 in the cortex (S1) after treatment with Cef for 15 days in APP/PS1 mice. Scale bar, 50 μm **(low)**. Scale bar, 10 μm **(high)**. **(H)** Quantification of AQP4 polarity after treatment with Cef for 15 days (*p* = 0.855, *t*-test, *n* = 4 mice per group). **(I)** Sample Western blots of brain MMP-9 levels after treatment with either XST **(upper)** or Cef **(lower)** in APP/PS1 mice. **(J)** Normalized levels of MMP-9 after treatment with XST (*p* = 0.003, *t*-test, *n* = 3 mice per group) or Cef (*p* = 0.390, *t*-test, *n* = 3 mice per group). **(K)** Quantitative measurement of VEGF-C levels in serum in WT and APP/PS1 mice (*p* = 0.6928, *t*-test, *n* = 6 mice per group). Data are mean ± SEM. *p < 0.05; **p < 0.01.

To further understand what could drive or underlie the observed distinct changes associated with XST or Cef treatment, we examined the levels of aquaporin 4 (AQP4) and glial glutamate transporter 1 (GLT-1) since they play important roles in regulating the exchange between brain parenchyma and CSF (Sharma et al., 2019; Simone et al., 2019). AQP4 could influence astrocytic calcium signaling (Yang et al., 2016) and potassium homeostasis and is associated with the removal of interstitial Aβ and modulation of glutamate-mediated synaptic transmission (Lan et al., 2017; Rasmussen et al., 2018) and AQP4 deficiency may also impair learning and memory, in part, through glutamate transporter-1 (GLT-1) (Lan et al., 2016a; Lan et al., 2016b). In addition, GLT-1 reduction and AQP4 alteration were reported in APP/PS1 mice (Takahashi et al., 2015; Benveniste et al., 2019). We found that XST treatment led to a significantly higher GLT-1 level but not AQP4 level in APP/PS1 mice (Figures 7A,B; *p* < 0.05). The polarity of AQP4 (see Methods for quantification) was significantly higher in APP/PS1 mice compared to WT mice (Figures 6C,D; *p* < 0.01), which reflects dysfunction of astrocytes (Lan et al., 2017). AQP4 polarity is significantly lower in the XST-treated than in Veh-treated APP/PS1 mice (Figures 7C,D; *p* < 0.05), a change that was absent in the WT mice. Similar to XST treatment, a significantly higher GLT-1 protein level but no change in AQP4 level was seen in APP/PS1 mice treated with Cef, compared to the Veh-treated group (Figures 7E,F, *p* < 0.05). However, AQP4 polarity was unchanged in Cef-injected APP/PS1 mice (Figures 7G,H). Since matrix metalloprotein 9 (MMP-9) has been implicated in targeting AQP4 to astrocyte endfeet (Yan et al., 2016; Amtul et al., 2018), we examined the level of MMP-9 in XST- or Cef-treated APP/PS1 mice. A significantly lower level of MMP-9 was seen only in the XST-treated group (Figures 7I,J, *p* < 0.01), suggesting reduced AQP4 polarity being mediated by reduced MMP-9 level. Furthermore, MMP-2/MMP-9 is known to regulate lymphangiogenesis (Du et al., 2017) and previous studies have found that VEGF significantly increases lymphangiogenesis (Wong et al., 2016). Moreover, hippocampus is an important brain area affected in AD. Thus, we also measured the protein levels of GLT1, AQP4, and MMP9 in the hippocampus and found changes similar to those at the whole-brain level (Supplementary Figures S2A,B). However, we found VEGF-C level not different between XST- and Veh-treated groups in the serum of APP/PS1 mice (Figure 7K), suggesting that VEGF-C-independent signaling pathway may underlie the XST-induced improvement in the lymphatic system.

## Discussion

Due to the unmet medical need in finding effective AD drugs, most studies aimed to identify new AD drugs by testing their efficacy in reducing pathology and improving functions in AD model animals. For effective drugs/compounds, elucidating the potential novel mechanism underlying their action is important for a better understanding of AD pathology and new therapies. Our previous study showed significant improvements in brain functions (spatial learning and memory, motor learning, and memory), enhanced CBF, and reduced amyloid plaque size and density in the AD transgenic mice after XST injections. Here, we focus on immune responses and potential changes in LVs associated with XST treatment. We found the following with XST injections: 1) enlarged LV structures; 2) increased clearance/removal of Aβ from LVs and lymph nodes and reduced amyloid plaque size and density in the brain (Huang et al., 2018; Mesquita et al., 2018); 3) higher level of anti-inflammatory cytokines (IL-1β and IL-10) and lower level of proinflammatory cytokine (IL-6) in both serum and brain; 4) an initial higher activation of microglia and astrocytes followed by reduced activation over the subsequent weeks to month. These results suggest a potential of XST to modulate the structure of the lymphatic system in a functionally meaningful setting, providing a better mechanistic understanding of the therapeutic impacts of XST on AD and a potential using small molecules to enhance the structure/function of LVs.

### Rescue of LV Deficits After XST Treatment

Meningeal LVs have recently been recognized as an important player in the complex circulation and exchange of soluble contents between CSF and ISF (Elham et al., 2020), and CSF-ISF exchange is a removal mechanism for macromolecules from the brain parenchyma to CSF (Jessen et al., 2015; Plog and Nedergaard, 2018). CSF circulation *via* the glymphatic system and meningeal LVs has been linked to neurological disorders (Rasmussen et al., 2018), including AD. Da Mesquita et al. (2018b) discovered that meningeal LVs drain macromolecules from the CNS (CSF and ISF) into the CLNs in mice. Aging-associated decrease in paravascular recirculation of CSF and ISF is likely to be responsible for the accumulation of Aβ in the brain parenchyma (Benveniste et al., 2019), and brain-CSF-dural LVs pathway is an alternative route of Aβ recirculation from brain ISF into the CSF sink (Sun et al., 2018b), supported by the presence of Aβ in the CLNs of AD transgenic mice (Karaman et al., 2018). Thus, an impaired meningeal lymphatic drainage of CSF may affect Aβ clearance and exacerbate amyloid burden in AD (He et al., 2017; Da Mesquita et al., 2018a). In addition, mice lacking meningeal LVs show dysfunction in their ability to clear parenchymal macromolecules (Aspelund et al., 2015; Ma et al., 2017). Our current findings of significant Aβ deposition in the meningeal lymphatics of APP/PS1 mice and significantly lower Aβ level in CLNs and meningeal LVs in XST-treated APP/PS1 mice are consistent with these findings. Hence, XST may increase meningeal LVs to remove Aβ from the CSF of APP/PS1 mice. Previous reports have shown that upregulation of dural lymphangiogenesis is associated with significantly reduced senile amyloid plaques in APP/PS1 mice (Karaman et al., 2018; Da Mesquita et al., 2018b), consistent with our finding, whereas we do not have direct evidence for enhanced functions of the LV system or direct evidence for removal of Aβ which is most directly revealed by imaging removal of injected fluorescent Aβ into the brain or real-time monitoring of Aβ clearance before and after XST administration. Due to technical reasons, we were not able to do this reliably. However, both our previous and current results have shown significant reduction in Aβ level or plaque size in the APP/PS1 mouse brains after XST treatment and in reducing levels of both fibrillar and surrounding soluble Aβ (Huang et al., 2018). Thus, we suggest that the improved clearance of Aβ may be related to the improved structures of LVs. In light of the ongoing debate on the functional contribution of LVs (Carare et al., 2020), we need to interpret our results with caution and need more direct examination to confirm the above hypothesis.

The newly discovered meningeal LVs are a novel path for cerebrospinal fluid drainage and represent a more conventional path for immune cells to egress the central nervous system (Louveau et al., 2015; Thomas et al., 2019). We found a significantly higher density of T-cells (CD3e^+^) in meningeal LVs of XST-treated APP/PS1 mice. In addition, IL-10 level was significantly increased and IL-6 level significantly decreased in the XST-treated group, consistent with reducing inflammation by XST (Da Mesquita et al., 2018b; Wang et al., 2019). We found that XST reduces the inflammatory responses in the serum and brains of transgenic AD mice, and this effect may be the result of improvements in LVs. Due to the potential multitarget effect of XST, it is quite possible that the modulation of inflammation is at least partially mediated by targets other than LVs. Reduced activation of microglia by XST may also ameliorate the production of proinflammatory cytokines, nitric oxide, and reactive oxygen species caused by excessive activation of microglia (Dando et al., 2018). It remains to be tested whether the initial elevated immune responses are a trigger for reducing Aβ level which results in subsequently reduced inflammation.

### How Does XST Improve the Lymphatic System?

One possibility is that XST increases the proliferation or activity of endothelial cells lining up the LVs. An alternative possibility is the improved LVs as an indirect effect of enhanced CBF. Many signal pathways are known to proliferate lymphatic endothelial cells and improve the lymphatic system function, including growth factors (VEGF, FGF, IGF, etc.) (Ren et al., 2017), Notch1/Akt (Blesinger et al., 2018; Sun et al., 2018a), MAPK-ERK1/2, and Wnt/β-catenin pathway (Zhao et al., 2017; Cong et al., 2018). However, we did not find the VEGF-C level altered by XST treatment. One major *in vivo* effect of XST is elevated CBF in both AD and WT mice (Huang et al., 2018), and this efficacy has been the main rationale for XST’s medical use in China (Du et al., 2015; Huang et al., 2018). Interestingly, Cef injection did not modulate LV structure and function albeit it significantly enhances CBF (Holschneider et al., 2020). Hence, despite the numerous and complex interactions between CBF and LVs (Kisler et al., 2017), directly enhancing CBF may not affect LVs, suggesting segregation of these two important fluid exchange systems in the brain. This is important not only for understanding the neurodegenerative diseases but also for development of successful therapeutic drugs. In addition, although both XST and Cef treatment lead to reduced inflammation in AD mice after 15 days, their paths are different: XST results in an initial activation of microglia followed by a decrease while Cef only leads to a significant decrease. Interestingly, XST results in a significant reduction in amyloid plaque level while Cef does not (Zumkehr et al., 2015; Fan et al., 2018).

Matrix metalloproteinase-9 (MMP-9) has been implicated in molecular mechanisms of AD (Kaminari et al., 2018), and MMP-9 expression plays an important role in excessive Aβ deposition (Ning et al., 2014). In addition, MMP-2/-9-mediated dystroglycan cleavage in astrocytes is likely to be crucial for maintaining the polarization of AQP4 (Yan et al., 2016; Zhang et al., 2018). The high expression of MMP-9 in the APP/PS1 mice (Kaminari et al., 2018; García-González et al., 2019) likely drives AQP4 to accumulate around amyloid plaques, hence showing high polarity. Glymphatic clearance mechanisms were strongly associated with AQP4 (Yin et al., 2018), and deletion of AQP4 in AD transgenic mice resulted in increased Aβ burden and exacerbated cognitive impairment (Rasmussen et al., 2018). Thus, reduced MMP expression by XST may contribute to the low polarity of AQP4, while Cef (which does not affect MMP-9 level) does not have a similar effect. GLT-1 (EAAT2 in human), the dominant glutamate transporter in the cerebral cortex and hippocampus, is significantly reduced in AD patients and mice (Pereira et al., 2017; Sharma et al., 2019), and increased GLT-1 level by Cef treatment was associated with improved cognitive ability in APP/PS1 mice (Fan et al., 2018). Thus, it is possible that the observed improvement in cognitive functions in XST- or Cef-treated AD mice is at least partially mediated by increased GLT-1 level.

### XST as Potential AD Drug by Improving Functions of LVs

Here, we provided substantial evidence for a widely used Chinese herbal medicine with the potential of improving LVs in AD model mice. Although significant changes have been observed on XST’s impact on BVs and LVs compared to the Veh-treated group, most of the improvements fell short of reversing to the level in the control/WT mice. This could be due to the following: 1) The treatment dose and duration which are not optimal. We have only tested one dose of XST which has been shown in improving cognitive functions and ameliorated AD pathology in APP/PS1 mice (Huang et al., 2018). It is possible that longer treatment may lead to better improvement. 2) The age of the AD mice. We have used AD mice at 11 months old which have developed prominent pathology and impaired functions. Although these mice are more likely to resemble AD patients when they receive medical treatment, the room for improvement is likely to be small. Future studies may test whether dosing XST in younger AD mice or with less advanced AD pathology may result in larger improvement. 3) Limited efficacy of XST as a drug on LV functions. Like other natural products, XST is not expected to exhibit high potency without significant chemical modification on a given biological function, and identification of key components in XST for improved efficacy and testing potential therapeutic path forward may be of interest.

## Conclusion

In summary, the interesting finding of the current study is that XST effectively increases the size of LVs and improves Aβ clearance ability of lymphatic systems. There is a potential to further develop for XST aiming to treat AD *via* enhancing LVs. Modulation of immune cells in the LVs and inflammatory cytokine levels *via* small molecules that can improve meningeal lymphatic functions might represent a novel and underexplored mechanism to improve brain functions, especially in the context of aging and neurodegeneration.

## Data Availability

The original contributions presented in the study are included in the article/Supplementary Material; further inquiries can be directed to the corresponding author.

## References

[B1] Ahn K. C. Learman C. R. Dunbar G. L. Maiti P. Jang W. C. Cha H. C. (2018). Characterization of Impaired Cerebrovascular Structure in APP/PS1 Mouse Brains. Neuroscience 385, 246–254. 10.1016/j.neuroscience.2018.05.002 29777753

[B2] Amtul Z. Yang J. Nikolova S. Lee T. Y. Bartha R. Cechetto D. F. (2018). The Dynamics of Impaired Blood-Brain Barrier Restoration in a Rat Model of Co-morbid Injury. Mol. Neurobiol. 55 (10), 8071–8083. 10.1007/s12035-018-0904-4 29508280

[B3] Aspelund A. Antila S. Proulx S. T. Karlsen T. V. Karaman S. Detmar M. (2015). A Dural Lymphatic Vascular System that Drains Brain Interstitial Fluid and Macromolecules. J. Exp. Med. 212 (7), 991–999. 10.1084/jem.20142290 26077718 PMC4493418

[B4] Aspelund A. Robciuc M. R. Karaman S. Makinen T. Alitalo K. (2016). Lymphatic System in Cardiovascular Medicine. Circ. Res. 118 (3), 515–530. 10.1161/circresaha.115.306544 26846644

[B5] Bálint L. Ocskay Z. Deák B. A. Aradi P. Jakus Z. (2019). Lymph Flow Induces the Postnatal Formation of Mature and Functional Meningeal Lymphatic Vessels. Front. Immunol. 10, 3043. 10.3389/fimmu.2019.03043 31993056 PMC6970982

[B6] Benveniste H. Liu X. Koundal S. Sanggaard S. Lee H. Wardlaw J. (2019). The Glymphatic System and Waste Clearance with Brain Aging: A Review. Gerontology 65 (2), 106–119. 10.1159/000490349 29996134 PMC6329683

[B7] Blesinger H. Kaulfuß S. Aung T. Schwoch S. Prantl L. Rößler J. (2018). PIK3CA Mutations Are Specifically Localized to Lymphatic Endothelial Cells of Lymphatic Malformations. PLoS One 13 (7), e0200343. 10.1371/journal.pone.0200343 29985963 PMC6037383

[B8] Bracko O. Njiru B. N. Swallow M. Ali M. Haft-Javaherian M. Schaffer C. B. (2019). Increasing Cerebral Blood Flow Improves Cognition into Late Stages in Alzheimer's Disease Mice. J. Cereb. Blood Flow Metab. 40, 1441–1452. 10.1177/0271678x19873658 31495298 PMC7308509

[B9] Brouillard P. Boon L. Vikkula M. (2014). Genetics of Lymphatic Anomalies. J. Clin. Invest. 124 (3), 898–904. 10.1172/jci71614 24590274 PMC3938256

[B10] Carare R. O. Aldea R. Agarwal N. Bacskai B. J. Bechman I. Boche D. (2020). Clearance of Interstitial Fluid (ISF) and CSF (CLIC) Group-Part of Vascular Professional Interest Area (PIA): Cerebrovascular Disease and the Failure of Elimination of Amyloid-β from the Brain and Retina with Age and Alzheimer's Disease-Opportunities for Therapy. Alzheimers Dement (Amst) 12 (1), e12053. 10.1002/dad2.12053 32775596 PMC7396859

[B11] Chen W. Huang P. Zeng H. Lin J. Shi Z. Yao X. (2020). Cocaine-induced Structural and Functional Impairments of the Glymphatic Pathway in Mice. Brain Behav. Immun. 88, 97–104. 10.1016/j.bbi.2020.04.057 32335199

[B12] Cong L. Zhang Y. Huang H. Cao J. Fu X. (2018). DFMG Reverses Proliferation and Migration of Vascular Smooth Muscle Cells Induced by Co-culture with Injured Vascular Endothelial Cells via Suppression of the TLR4-Mediated Signaling Pathway. Mol. Med. Rep. 17 (4), 5692–5699. 10.3892/mmr.2018.8635 29484442 PMC5866011

[B13] Da Mesquita S. Fu Z. Kipnis J. (2018a). The Meningeal Lymphatic System: A New Player in Neurophysiology. Neuron 100 (2), 375–388. 10.1016/j.neuron.2018.09.022 30359603 PMC6268162

[B14] Da Mesquita S. Louveau A. Vaccari A. Smirnov I. Cornelison R. C. Kingsmore K. M. (2018b). Functional Aspects of Meningeal Lymphatics in Ageing and Alzheimer's Disease. Nature 560 (7717), 185–191. 10.1038/s41586-018-0368-8 30046111 PMC6085146

[B15] Da Mesquita S. Louveau A. Vaccari A. Smirnov I. Cornelison R. C. Kingsmore K. M. (2018). Functional Aspects of Meningeal Lymphatics in Ageing and Alzheimer's Disease. Nature 560 (15), 185–191. 10.1038/s41586-018-0368-8 30046111 PMC6085146

[B16] Dando S. J. Kazanis R. Chinnery H. R. McMenamin P. G. (2018). Regional and Functional Heterogeneity of Antigen Presenting Cells in the Mouse Brain and Meninges. Glia 67 (8), 935–949. 10.1002/glia.23581 30585356

[B17] Dave R. S. Jain P. Byrareddy S. N. (2018). Functional Meningeal Lymphatics and Cerebrospinal Fluid Outflow. J. Neuroimmune Pharmacol. 13 (2), 123–125. 10.1007/s11481-018-9778-5 29464588 PMC5930060

[B18] Du H. T. Du L. L. Tang X. L. Ge H. Y. Liu P. (2017). Blockade of MMP-2 and MMP-9 Inhibits Corneal Lymphangiogenesis. Graefes Arch. Clin. Exp. Ophthalmol. 255 (8), 1573–1579. 10.1007/s00417-017-3651-8 28669039

[B19] Du R. Teng J. F. Wang Y. Zhao X. Y. Shi Z. B. (2015). Clinical Study of Butylphthalide Combined with Xue Shuan Tong on Serum Inflammatory Factors and Prognosis Effect of Patients with Cerebral Infarction. Pak J. Pharm. Sci. 28 (5 Suppl. l), 1823–1827. 26525022

[B20] Elham E. Wumaier R. Wang C. Luo X. Chen T. Zhong N. (2020). Anatomic Evidence Shows that Lymphatic Drainage Exists in the Pituitary to Loop the Cerebral Lymphatic Circulation. Med. Hypotheses 143, 109898. 10.1016/j.mehy.2020.109898 32504926 PMC7260572

[B21] Fakhoury M. (2018). Microglia and Astrocytes in Alzheimer's Disease: Implications for Therapy. Curr. Neuropharmacol 16 (5), 508–518. 10.2174/1570159x15666170720095240 28730967 PMC5997862

[B22] Fan S. Xian X. Li L. Yao X. Hu Y. Zhang M. (2018). Ceftriaxone Improves Cognitive Function and Upregulates GLT-1-Related Glutamate-Glutamine Cycle in APP/PS1 Mice. J. Alzheimers Dis. 66 (4), 1731–1743. 10.3233/jad-180708 30452416

[B23] Gao X. Zheng R. Ma X. Gong Z. Xia D. Zhou Q. (2019). Elevated Level of PKMζ Underlies the Excessive Anxiety in an Autism Model. Front. Mol. Neurosci. 12, 291. 10.3389/fnmol.2019.00291 31849605 PMC6893886

[B24] García-González L. Pilat D. Baranger K. Rivera S. (2019). Emerging Alternative Proteinases in APP Metabolism and Alzheimer's Disease Pathogenesis: A Focus on MT1-MMP and MT5-MMP. Front. Aging Neurosci. 11, 244. 10.3389/fnagi.2019.00244 31607898 PMC6769103

[B25] Guimarães-Souza E. M. Joselevitch C. Britto L. R. G. Chiavegatto S. (2019). Retinal Alterations in a Pre-clinical Model of an Autism Spectrum Disorder. Mol. Autism 10, 19. 10.1186/s13229-019-0270-8 31011411 PMC6466731

[B26] Guo H. Adah D. James P. B. Liu Q. Li G. Ahmadu P. (2018). Xueshuantong Injection (Lyophilized) Attenuates Cerebral Ischemia/Reperfusion Injury by the Activation of Nrf2-VEGF Pathway. Neurochem. Res. 43 (5), 1096–1103. 10.1007/s11064-018-2523-x 29633164

[B27] He Z. F. Chen J. Zhou C. N. Rao Z. Wang X. H. (2017). Disabling Tremor Induced by Long-Term Use of Sodium Valproate and Lamotrigine: Case Report. Medicine (Baltimore) 96 (47), e8711. 10.1097/MD.0000000000008711 29381960 PMC5708959

[B28] Holschneider D. P. Wang Z. Chang H. Zhang R. Gao Y. Guo Y. (2020). Ceftriaxone Inhibits Stress‐induced Bladder Hyperalgesia and Alters Cerebral Micturition and Nociceptive Circuits in the Rat: A Multidisciplinary Approach to the Study of Urologic Chronic Pelvic Pain Syndrome Research Network Study. Neurourology and Urodynamics 39, 1628–1643. 10.1002/nau.24424 32578247 PMC7642011

[B29] Huang Y. Guo B. Shi B. Gao Q. Zhou Q. (2018). Chinese Herbal Medicine Xueshuantong Enhances Cerebral Blood Flow and Improves Neural Functions in Alzheimer's Disease Mice. J. Alzheimers Dis. 63 (3), 1089–1107. 10.3233/jad-170763 29710701 PMC6004915

[B30] Jessen N. A. Munk A. S. Lundgaard I. Nedergaard M. (2015). The Glymphatic System: A Beginner's Guide. Neurochem. Res. 40 (12), 2583–2599. 10.1007/s11064-015-1581-6 25947369 PMC4636982

[B31] Kametani F. Hasegawa M. (2018). Reconsideration of Amyloid Hypothesis and Tau Hypothesis in Alzheimer's Disease. Front. Neurosci. 12, 25. 10.3389/fnins.2018.00025 29440986 PMC5797629

[B32] Kaminari A. Tsilibary E. C. Tzinia A. (2018). A New Perspective in Utilizing MMP-9 as a Therapeutic Target for Alzheimer's Disease and Type 2 Diabetes Mellitus. J. Alzheimers Dis. 64 (1), 1–16. 10.3233/jad-180035 29865065

[B33] Karaman S. Leppänen V. M. Alitalo K. (2018). Vascular Endothelial Growth Factor Signaling in Development and Disease. Development 145 (14). 10.1242/dev.151019 30030240

[B34] Kaur D. Sharma V. Deshmukh R. (2019). Activation of Microglia and Astrocytes: a Roadway to Neuroinflammation and Alzheimer's Disease. Inflammopharmacology 27 (4), 663–677. 10.1007/s10787-019-00580-x 30874945

[B35] Kim K. W. Song J. H. (2017). Emerging Roles of Lymphatic Vasculature in Immunity. Immune Netw. 17 (1), 68–76. 10.4110/in.2017.17.1.68 28261022 PMC5334124

[B36] Kisler K. Nelson A. R. Montagne A. Zlokovic B. V. (2017). Cerebral Blood Flow Regulation and Neurovascular Dysfunction in Alzheimer Disease. Nat. Rev. Neurosci. 18 (7), 419–434. 10.1038/nrn.2017.48 28515434 PMC5759779

[B37] Klotz L. Norman S. Vieira J. M. Masters M. Rohling M. Dubé K. N. (2015). Cardiac Lymphatics Are Heterogeneous in Origin and Respond to Injury. Nature 522 (7554), 62–67. 10.1038/nature14483 25992544 PMC4458138

[B38] Koronyo-Hamaoui M. Ko M. K. Koronyo Y. Azoulay D. Seksenyan A. Kunis G. (2009). Attenuation of AD-like Neuropathology by Harnessing Peripheral Immune Cells: Local Elevation of IL-10 and MMP-9. J. Neurochem. 111 (6), 1409–1424. 19780903 10.1111/j.1471-4159.2009.06402.x

[B39] Kortela E. Hytönen J. Numminen J. Overmyer M. Saxen H. Oksi J. (2017). Cerebral Vasculitis and Intracranial Multiple Aneurysms in a Child with Lyme Neuroborreliosis. JMM Case Rep. 4 (4), e005090. 10.1099/jmmcr.0.005090 29026617 PMC5630958

[B40] Kyrtsos C. Baras J. (2014). “The Glymphatic System and Alzheimer’s Disease: Possible Connection? ”. The Sixth International Conference on Bioinformatics, Biocomputational Systems and Biotechnologies.

[B41] Lan Y. L. Chen J. J. Hu G. Xu J. Xiao M. Li S. (2017). Aquaporin 4 in Astrocytes Is a Target for Therapy in Alzheimer's Disease. Curr. Pharm. Des. 23 (33), 4948–4957. 10.2174/1381612823666170714144844 28714415

[B42] Lan Y. L. Zhao J. Ma T. Li S. (2016a). The Potential Roles of Aquaporin 4 in Alzheimer's Disease. Mol. Neurobiol. 53 (8), 5300–5309. 10.1007/s12035-015-9446-1 26433375

[B43] Lan Y. L. Zou S. Chen J. J. Zhao J. Li S. (2016b). The Neuroprotective Effect of the Association of Aquaporin-4/Glutamate Transporter-1 against Alzheimer's Disease. Neural Plast. 2016, 4626593. 10.1155/2016/4626593 27057365 PMC4736756

[B44] Li R. L. Wang J. X. Chai L. J. Guo H. Wang H. Chen L. (2020). Xueshuantong for Injection (Lyophilized, ) Alleviates Streptozotocin-Induced Diabetic Retinopathy in Rats. Chin. J. Integr. Med. 26 (11), 825–832. 10.1007/s11655-020-3088-5 32415646

[B45] Louveau A. Plog B. A. Antila S. Alitalo K. Nedergaard M. Kipnis J. (2017). Understanding the Functions and Relationships of the Glymphatic System and Meningeal Lymphatics. J. Clin. Invest. 127 (9), 3210–3219. 10.1172/jci90603 28862640 PMC5669566

[B46] Louveau A. Smirnov I. Keyes T. J. Eccles J. D. Rouhani S. J. Peske J. D. (2015). Structural and Functional Features of central Nervous System Lymphatic Vessels. Nature 523 (7560), 337–341. 10.1038/nature14432 26030524 PMC4506234

[B47] Ma Q. Ineichen B. V. Detmar M. Proulx S. T. (2017). Outflow of Cerebrospinal Fluid Is Predominantly through Lymphatic Vessels and Is Reduced in Aged Mice. Nat. Commun. 8 (1), 1434. 10.1038/s41467-017-01484-6 29127332 PMC5681558

[B48] Ning M. Wu Y. Ni X. Zhao Y. Ling R. (2014). Cerebral Microemboli Increase β-amyloid Protein Deposition, MMP-9, and GFAP Expression in the Alzheimer`s Model of APP/PS1 Double Transgenic Mice. Curr. Neurovasc Res. 11 (3), 190–201. 10.2174/1567202611666140606104132 24962158

[B49] Patel A. Simon S. M Elangoven I. Amalchandran J. S Jain T. (2019). Dopamine Transporter Maging with Tc-99m TRODAT-1 SPECT in Parkinson's Isease and its Orrelation with Linical Isease Everity. Asia Ocean J. Nucl. Med. Biol. 7 (1), 22–28. 10.22038/AOJNMB.2018.30356.1208 30705908 PMC6352051

[B50] Pereira A. C. Gray J. D. Kogan J. F. Davidson R. L. Rubin T. G. Okamoto M. (2017). Age and Alzheimer's Disease Gene Expression Profiles Reversed by the Glutamate Modulator Riluzole. Mol. Psychiatry 22 (2), 296–305. 10.1038/mp.2016.33 27021815 PMC5042881

[B51] Plog B. A. Nedergaard M. (2018). The Glymphatic System in Central Nervous System Health and Disease: Past, Present, and Future. Annu. Rev. Pathol. 13, 379–394. 10.1146/annurev-pathol-051217-111018 29195051 PMC5803388

[B52] Polanco J. C. Li C. Bodea L. G. Martinez-Marmol R. Meunier F. A. Götz J. (2018). Amyloid-β and Tau Complexity - towards Improved Biomarkers and Targeted Therapies. Nat. Rev. Neurol. 14 (1), 22–39. 10.1038/nrneurol.2017.162 29242522

[B53] Rasmussen M. K. Mestre H. Nedergaard M. (2018). The Glymphatic Pathway in Neurological Disorders. Lancet Neurol. 17 (11), 1016–1024. 10.1016/s1474-4422(18)30318-1 30353860 PMC6261373

[B54] Ren J. G. Xia H. F. Yang J. G. Zhu J. Y. Zhang W. Chen G. (2017). Down-regulation of Polycystin in Lymphatic Malformations: Possible Role in the Proliferation of Lymphatic Endothelial Cells. Hum. Pathol. 65, 231–238. 10.1016/j.humpath.2017.05.016 28552828

[B55] Schläger C. Körner H. Krueger M. Vidoli S. Haberl M. Mielke D. (2016). Effector T-Cell Trafficking between the Leptomeninges and the Cerebrospinal Fluid. Nature 530 (7590), 349–353. 10.1038/nature16939 26863192

[B56] Sharma A. Kazim S. F. Larson C. S. Ramakrishnan A. Gray J. D. McEwen B. S. (2019). Divergent Roles of Astrocytic versus Neuronal EAAT2 Deficiency on Cognition and Overlap with Aging and Alzheimer's Molecular Signatures. Proc. Natl. Acad. Sci. U S A. 116 (43), 21800–21811. 10.1073/pnas.1903566116 31591195 PMC6815169

[B57] Shechter R. London A. Schwartz M. (2013). Orchestrated Leukocyte Recruitment to Immune-Privileged Sites: Absolute Barriers versus Educational gates. Nat. Rev. Immunol. 13 (3), 206–218. 10.1038/nri3391 23435332

[B58] Simone L. Pisani F. Mola M. G. De Bellis M. Merla G. Micale L. (2019). AQP4 Aggregation State Is a Determinant for Glioma Cell Fate. Cancer Res. 79 (9), 2182–2194. 10.1158/0008-5472.Can-18-2015 30877104

[B59] Sun B. Dong C. Lei H. Gong Y. Li M. Zhang Y. (2018a). Propranolol Inhibits Proliferation and Invasion of Hemangioma-Derived Endothelial Cells by Suppressing the DLL4/Notch1/Akt Pathway. Chem. Biol. Interact 294, 28–33. 10.1016/j.cbi.2018.08.018 30130526

[B60] Sun B. L. Wang L. H. Yang T. Sun J. Y. Mao L. L. Yang M. F. (2018b). Lymphatic Drainage System of the Brain: A Novel Target for Intervention of Neurological Diseases. Prog. Neurobiol. 163-164, 118–143. 10.1016/j.pneurobio.2017.08.007 28903061

[B61] Takahashi K. Foster J. B. Lin C. L. (2015). Glutamate Transporter EAAT2: Regulation, Function, and Potential as a Therapeutic Target for Neurological and Psychiatric Disease. Cell Mol Life Sci 72 (18), 3489–3506. 10.1007/s00018-015-1937-8 26033496 PMC11113985

[B62] Tarasoff-Conway J. M. Carare R. O. Osorio R. S. Glodzik L. Butler T. Fieremans E. (2016). Clearance Systems in the Brain-Iimplications for Alzheimer Diseaser. Nat. Rev. Neurol. 12 (4), 248. 10.1038/nrneurol.2016.36 27020556

[B63] Thomas J. L. Jacob L. Boisserand L. (2019). [Lymphatic System in central Nervous System]. Med. Sci. (Paris) 35 (1), 55–61. 10.1051/medsci/2018309 30672459

[B64] Toly-Ndour C. Lui G. Nunes M. M. Bruley-Rosset M. Aucouturier P. Dorothée G. (2011). MHC-independent Genetic Factors Control the Magnitude of CD4+ T Cell Responses to Amyloid-β Peptide in Mice through Regulatory T Cell-Mediated Inhibition. J. Immunol. 187 (9), 4492–4500. 10.4049/jimmunol.1003953 21949026

[B65] van der Kleij L. A. Petersen E. T. Siebner H. R. Hendrikse J. Frederiksen K. S. Sobol N. A. (2018). The Effect of Physical Exercise on Cerebral Blood Flow in Alzheimer's Disease. Neuroimage Clin. 20, 650–654. 10.1016/j.nicl.2018.09.003 30211001 PMC6129739

[B66] Venero Galanternik M. Stratman A. N. Jung H. M. Butler M. G. Weinstein B. M. (2016). Building the Drains: The Lymphatic Vasculature in Health and Disease. Wiley Interdiscip. Rev. Dev. Biol. 5 (6). 10.1002/wdev.246 27576003

[B67] Vogelsang P. Giil L. M. Lund A. Vedeler C. A. Parkar A. P. Nordrehaug J. E. (2018). Reduced Glucose Transporter-1 in Brain Derived Circulating Endothelial Cells in Mild Alzheimer's Disease Patients. Brain Res. 1678, 304–309. 10.1016/j.brainres.2017.10.035 29102777

[B68] Wang F. J. Wang S. X. Chai L. J. Zhang Y. Guo H. Hu L. M. (2018). Xueshuantong Injection (Lyophilized) Combined with Salvianolate Lyophilized Injection Protects against Focal Cerebral Ischemia/reperfusion Injury in Rats through Attenuation of Oxidative Stress. Acta Pharmacol. Sin 39 (6), 998–1011. 10.1038/aps.2017.128 29022576 PMC6256270

[B69] Wang L. Zhang Y. Zhao Y. Marshall C. Wu T. Xiao M. (2019). Deep Cervical Lymph Node Ligation Aggravates AD-like Pathology of APP/PS1 Mice. Brain Pathol. 29 (2), 176–192. 10.1111/bpa.12656 30192999 PMC8028636

[B70] Wang M. Iliff J. J. Liao Y. Chen M. J. Shinseki M. S. Venkataraman A. (2012). Cognitive Deficits and Delayed Neuronal Loss in a Mouse Model of Multiple Microinfarcts. J. Neurosci. 32 (50), 17948–17960. 10.1523/jneurosci.1860-12.2012 23238711 PMC3541041

[B71] Wang X. Wang S. Wang J. Guo H. Dong Z. Chai L. (2015). Neuroprotective Effect of Xueshuantong for Injection (Lyophilized) in Transient and Permanent Rat Cerebral Ischemia Model. Evid. Based Complement. Alternat Med. 2015, 134685. 10.1155/2015/134685 26681963 PMC4670871

[B72] Williams A. S. Jones S. G. Goodfellow R. M. Amos N. Williams B. D. (1999). Interleukin-1beta (IL-1beta) Inhibition: a Possible Mechanism for the Anti-inflammatory Potency of Liposomally Conjugated Methotrexate Formulations in Arthritis. Br. J. Pharmacol. 128 (1), 234–240. 10.1038/sj.bjp.0702776 10498857 PMC1571613

[B73] Wong H. L. Jin G. Cao R. Zhang S. Cao Y. Zhou Z. (2016). MT1-MMP Sheds LYVE-1 on Lymphatic Endothelial Cells and Suppresses VEGF-C Production to Inhibit Lymphangiogenesis. Nat. Commun. 7, 10824. 10.1038/ncomms10824 26926389 PMC4773521

[B74] Yan W. Zhao X. Chen H. Zhong D. Jin J. Qin Q. (2016). β-Dystroglycan Cleavage by Matrix Metalloproteinase-2/-9 Disturbs Aquaporin-4 Polarization and Influences Brain Edema in Acute Cerebral Ischemia. Neuroscience 326, 141–157. 10.1016/j.neuroscience.2016.03.055 27038751

[B75] Yang C. Huang X. Huang X. Mai H. Li J. Jiang T. (2016). Aquaporin-4 and Alzheimer's Disease. J. Alzheimers Dis. 52 (2), 391–402. 10.3233/jad-150949 27031475

[B76] Yao C. Yang W. Zhang J. Qiu S. Chen M. Shi X. (2017). UHPLC-Q-TOF-MS-based Metabolomics Approach to Compare the Saponin Compositions of Xueshuantong Injection and Xuesaitong Injection. J. Sep. Sci. 40 (4), 834–841. 10.1002/jssc.201601122 27935213

[B77] Yin M. Pu T. Wang L. Marshall C. Wu T. Xiao M. (2018). Astroglial Water Channel Aquaporin 4-mediated Glymphatic Clearance Function: A Determined Factor for Time-Sensitive Treatment of Aerobic Exercise in Patients with Alzheimer's Disease. Med. Hypotheses 119, 18–21. 10.1016/j.mehy.2018.07.016 30122483

[B78] Zhang H. Y. Niu W. Olaleye O. E. Du F. F. Wang F. Q. Huang Y. H. (2020). Comparison of Intramuscular and Intravenous Pharmacokinetics of Ginsenosides in Humans after Dosing XueShuanTong, a Lyophilized Extract of Panax Notoginseng Roots. J. Ethnopharmacol 253, 112658. 10.1016/j.jep.2020.112658 32035876

[B79] Zhang Z. Zhang L. Chen J. Cao Y. Qu M. Lin X. (2018). 2-(2-Benzofuranyl)-2-Imidazoline Mediates Neuroprotection by Regulating the Neurovascular Unit Integrity in a Rat Model of Focal Cerebral Ischemia. J. Stroke Cerebrovasc. Dis. 27 (6), 1481–1489. 10.1016/j.jstrokecerebrovasdis.2017.12.041 29398538

[B80] Zhao B. Zhao Z. Sun X. Zhang Y. Guo Y. Tian P. (2017). Effect of Micro Strain Stress on Proliferation of Endothelial Progenitor Cells *In Vitro* by the MAPK-Erk1/2 Signaling Pathway. Biochem. Biophys. Res. Commun. 492 (2), 206–211. 10.1016/j.bbrc.2017.08.050 28821432

[B81] Zhu T. Zhang F. Li H. He Y. Zhang G. Huang N. (2019). Long-term Icariin Treatment Ameliorates Cognitive Deficits via CD4+ T Cell-Mediated Immuno-Inflammatory Responses in APP/PS1 Mice. Clin. Interv. Aging 14, 817–826. 10.2147/cia.S208068 31190768 PMC6511656

[B82] Zumkehr J. Rodriguez-Ortiz C. J. Cheng D. Kieu Z. Wai T. Hawkins C. (2015). Ceftriaxone Ameliorates Tau Pathology and Cognitive Decline via Restoration of Glial Glutamate Transporter in a Mouse Model of Alzheimer's Disease. Neurobiol. Aging 36 (7), 2260–2271. 10.1016/j.neurobiolaging.2015.04.005 25964214

