# Supplementary figures and images for: Xueshuantong Improves Functions of Lymphatic Ducts and Modulates Inflammatory Responses in Alzheimer’s Disease Mice
Source: Front Pharmacol. 2021 Sep 28;12:605814. doi: 10.3389/fphar.2021.605814 (PMC8505705; doi:10.3389/fphar.2021.605814)

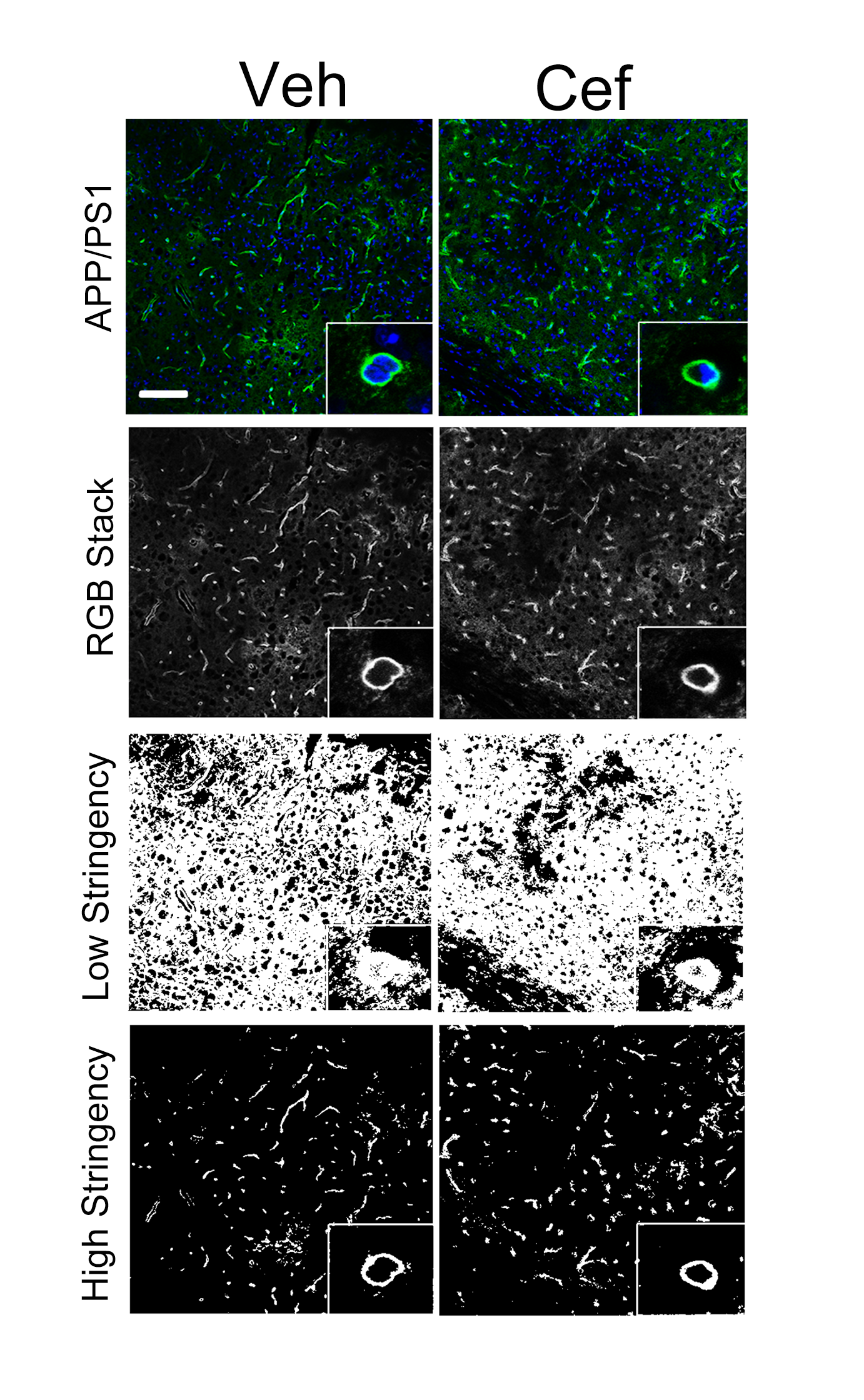

Supplement: Supplementary file 1 [file Image3.TIF]

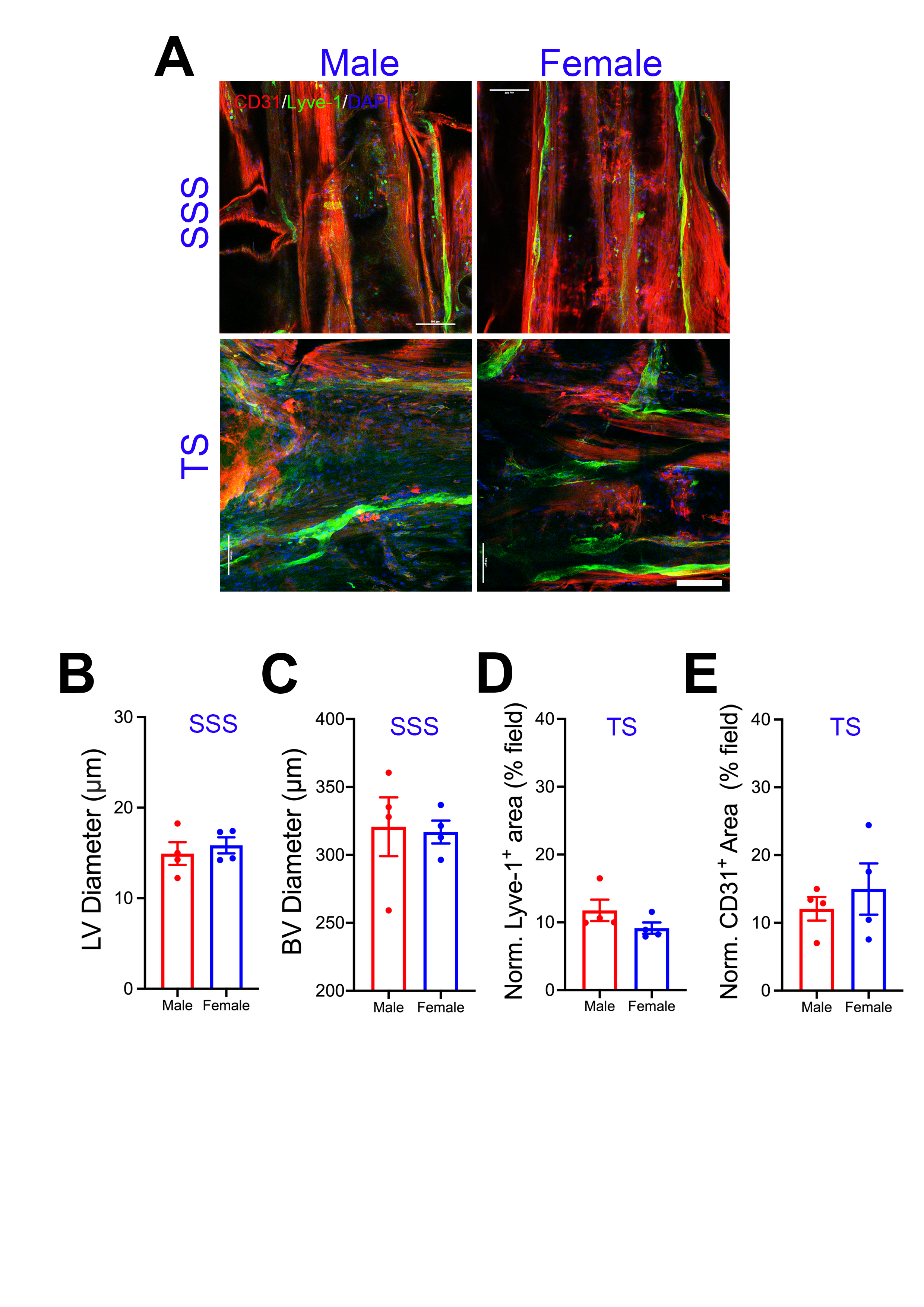

Supplement: Supplementary file 2 [file Image4.TIF]

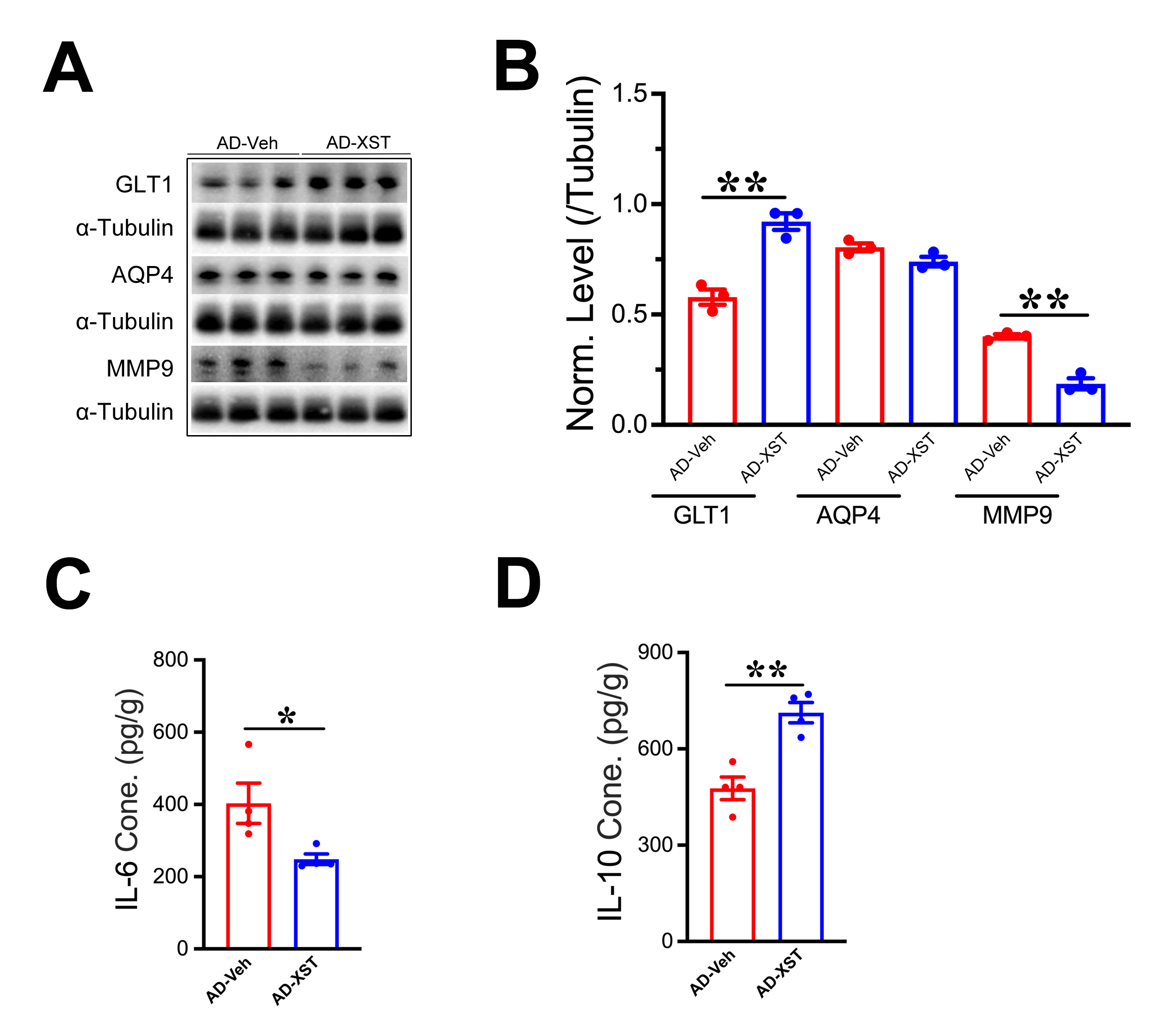

Supplement: Supplementary file 3 [file Image2.TIF]

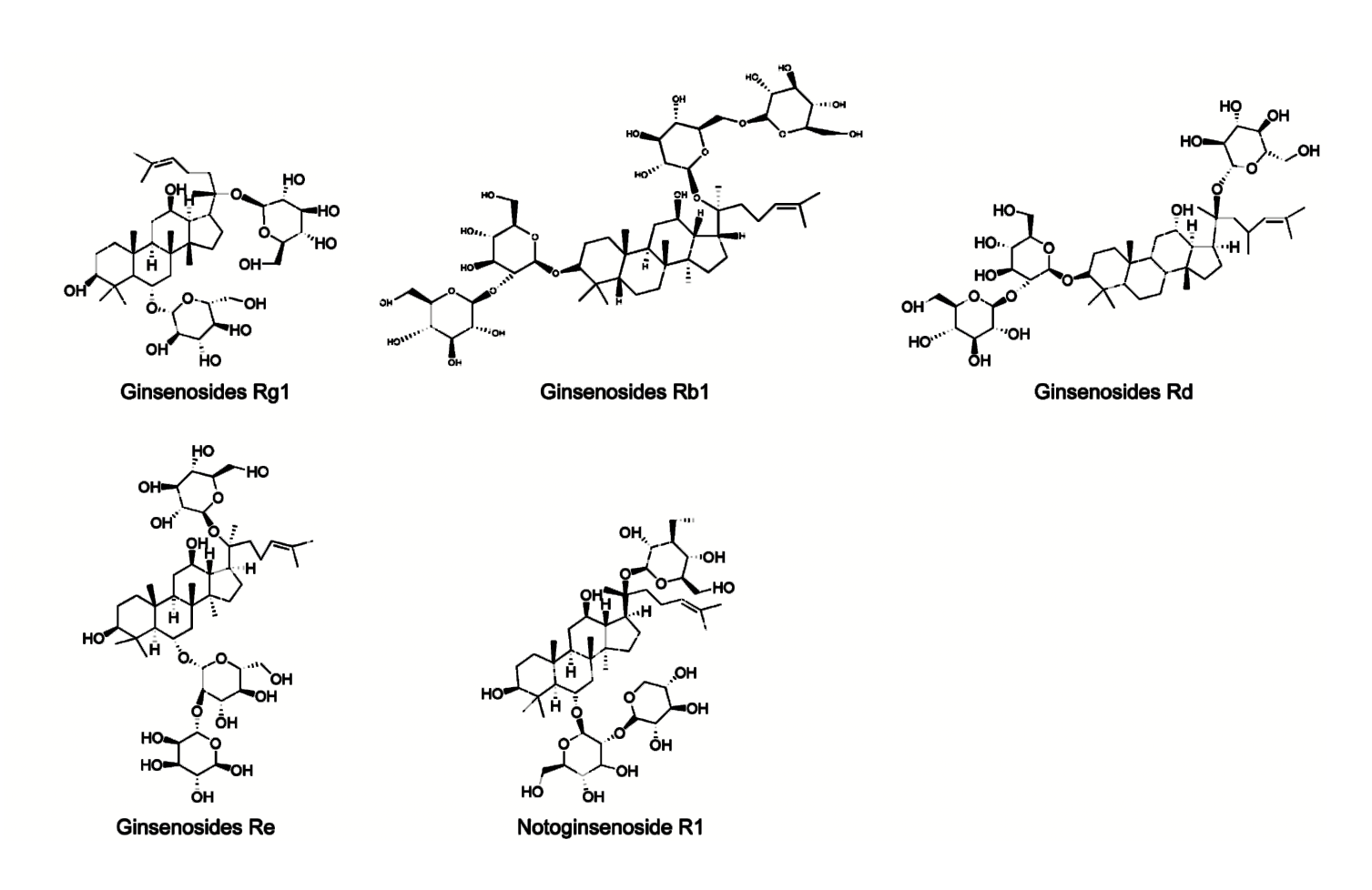

Supplement: Supplementary file 4 [file Image1.TIF]
